# Synergistic Modulating of Mitochondrial Transfer and Immune Microenvironment to Attenuate Discogenic Pain

**DOI:** 10.1002/advs.202500128

**Published:** 2025-03-27

**Authors:** Xinzhou Wang, Zhenyu Guo, Linjie Chen, Jing Sun, Kenny Yat Hong Kwan, Morgan Jones, Yan Michael Li, Yangyang Hu, Xueqiang Wang, Pooyan Makvandi, Xiangyang Wang, Qiuping Qian, Yunlong Zhou, Aimin Wu

**Affiliations:** ^1^ Department of Orthopaedics Key Laboratory of Structural Malformations in Children of Zhejiang Province Key Laboratory of Orthopaedics of Zhejiang Province Rehabilitation Medicine Center The Second Affiliated Hospital and Yuying Children's Hospital of Wenzhou Medical University Wenzhou 325000 China; ^2^ Zhejiang Engineering Research Center for Tissue Repair Materials Wenzhou Institute University of Chinese Academy of Sciences Wenzhou 325000 China; ^3^ Department of Orthopaedics and Traumatology Li Ka Shing Faculty of Medicine The University of Hong Kong Hong Kong SAR China; ^4^ Spine Unit The Royal Orthopaedic Hospital Birmingham B31 2AP UK; ^5^ Minimally Invasive Brain and Spine Institute State University of New York Upstate Medical University 475 Irving Ave, #402 Syracuse NY 13210 USA; ^6^ Department of Orthopedics The First Affiliated Hospital of Wenzhou Medical University Wenzhou 325000 China; ^7^ The Quzhou Affiliated Hospital of Wenzhou Medical University Quzhou People's Hospital Quzhou 324000 China

**Keywords:** discogenic pain, macrophage, mitochondrial transfer, nanoparticles

## Abstract

Discogenic pain, caused by intervertebral disc degeneration (IVDD), is a prevalent and challenging condition to treat effectively. Macrophage infiltration with neural ectopic in‐growth resulting from structural disturbances within the intervertebral disc (IVD) is a major cause of discogenic pain. This work systematically reveals how nanoparticles can synergistically regulate the immune microenvironment and mitochondrial communication to attenuate discogenic pain. The antioxidant metal‐polyphenol nanoparticle system can sequentially regulate macrophage phenotype and mitochondrial delivery efficiency. This strategy circumvents the necessity for mitochondrial isolation and preservation techniques that are typically required in conventional mitochondrial transplantation procedures. Furthermore, it facilitates the effective and sustained delivery of mitochondria to damaged cells. In vivo, this nanoparticle formulation effectively preserves the IVD height, maintains the structural integrity of the nucleus pulposus (NP), and restores pain thresholds. Thus, this nanoplatform offers an effective approach to traditional surgical treatments for discogenic pain, with significant potential for clinical application.

## Graphical abstract

Gallic acid (GA) and copper ions self‐assemble to form nanoparticles, which are then modified with mitochondrial targeting peptides and gap junction modulator. These nanoparticles scavenge mitochondrial reactive oxygen species to induce M2 polarization and enhance intercellular mitochondrial transfer. Within the intervertebral disc, macrophage‐derived healthy mitochondria efficiently maintain mitochondrial function in damaged cells to alleviate discogenic pain.

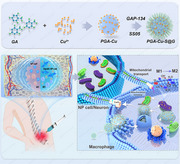

## 1 Introduction

According to the Global Burden of Disease study, low back pain (LBP) is the leading cause of disability and limitation of daily living in adults worldwide.^[^ ^1^ ^]^ It is predicted that more than 800 million people worldwide will suffer from LBP by 2050.^[^ ^2^ ^]^ As such, LBP represents a significant medical burden and a major global challenge to public health infrastructure.^[^ ^3^ ^]^ Although LBP is often considered non‐specific, the most common specific cause is IVDD, also known as discogenic pain.^[^ ^4^ ^]^ This pain is primarily the result of a disruption in the internal structure of the IVD. As IVDD progresses, the collagen fibers in the annulus fibrosus (AF) become disorganized, and the cartilage endplates (CE) undergo severe destruction and hardening.^[^ ^5^ ^]^ These structural changes compromise the physiological barrier of the NP, resulting in exposure to the immune system and macrophage infiltration, which tend to become M1‐polarized.^[^ ^6^ ^]^ Additionally, nerve fibers that were initially located outside the AF invade the NP.^[^ ^7^ ^]^ The inflammatory and neurotrophic factors secreted by M1 macrophages stimulate the expression of pain‐associated cation channels and the production of pain transmitters in the dorsal root ganglion, thereby developing discogenic pain.^[^ ^8^ ^]^ Consequently, a complex pathological microenvironment of multicellular coexistence is formed in the nucleus pulposus in the IVDD state.

Most previous studies have concentrated on the function of individual cell types in the context of IVDD,^[^ ^9^ ^]^ with a notable absence of research examining the collective influence of intercellular interactions on disc microenvironmental homeostasis. It is crucial to acknowledge that IVD is a highly organized and dynamic tissue. The disruption of one cell type can have a cascading effect on other cell types, leading to the onset of multifaceted degenerative processes. In particular, the interaction of immune cells with histiocytes is more complex than that of other cell types, and this area remains under‐explored. In IVDD, an imbalance in macrophage polarization results in significant oxidative stress on NP cells and neurons.^[^ ^10^ ^]^ Oxidative stress plays a pivotal role in mitochondrial damage, which is the site of cellular redox reactions. Impaired mitochondrial function results in cellular dysfunction and reduced activity.^[^ ^11^ ^]^ Therefore, it is imperative to investigate the potential of structured therapeutic approaches within a disrupted pathological environment. Mitochondrial transfer represents an emerging therapeutic strategy for mitochondrial complementation. This approach involves mitochondria transfer between cells in a non‐vertical genetic manner. It has been demonstrated to be an effective means of restoring mitochondrial homeostasis.^[^ ^12^ ^]^ In contrast to direct replenishment, mitochondrial transfer leverages cells' intrinsic capacity to transfer mitochondria spontaneously, obviating the necessity for isolation and preservation.^[^ ^13^ ^]^ Multiple investigations have substantiated that macrophages can serve as donor cells to furnish healthy mitochondria to injured cells.^[^ ^14^ ^]^ Macrophages are pervasively distributed, communicate extensively with neighboring cells, and can migrate to lesions, thus facilitating mitochondrial delivery.^[^ ^15^ ^]^ Given the dual role of macrophages in IVDD, we propose a potential therapeutic strategy to convert macrophage polarization ratio from M1‐type to M2‐type. Subsequently, M2 macrophages could rescue damaged cells via mitochondrial transfer, thereby alleviating discogenic pain through mechanisms beyond purely anti‐inflammatory effects.

Nevertheless, the sustained and effective delivery of mitochondria to damaged cells represents a significant challenge. One of the primary limitations to the efficacy of mitochondrial transfer is the quality of mitochondria in donor cells.^[^ ^16^ ^]^ Reactive oxygen species (ROS), a byproduct of mitochondrial metabolism, significantly contributes to mitochondrial dysfunction. Additionally, ROS, especially mitochondria‐derived reactive oxygen species (mtROS) plays a pivotal role in macrophage polarization, inducing M1‐type and inhibiting M2‐type polarization.^[^ ^17^ ^]^ Consequently, removing mtROS from macrophage can restore mitochondrial function and shift macrophage polarization from M1‐type to M2‐type, thereby improving the quality of mitochondrial delivery from macrophages. Moreover, the efficacy of mitochondrial transfer is constrained by its transfer efficiency.^[^ ^18^ ^]^ Tunneling nanotubes (TNTs) represent a pivotal pathway for mitochondrial transfer. Functional gap junction channels (GJCs) constitute TNTs extension terminals that regulate TNTs formation and stability.^[^ ^19^ ^]^ The up‐regulation of gap junction proteins (e.g., Cx43) effectively enhances transfer efficiency.

**Scheme 1:**
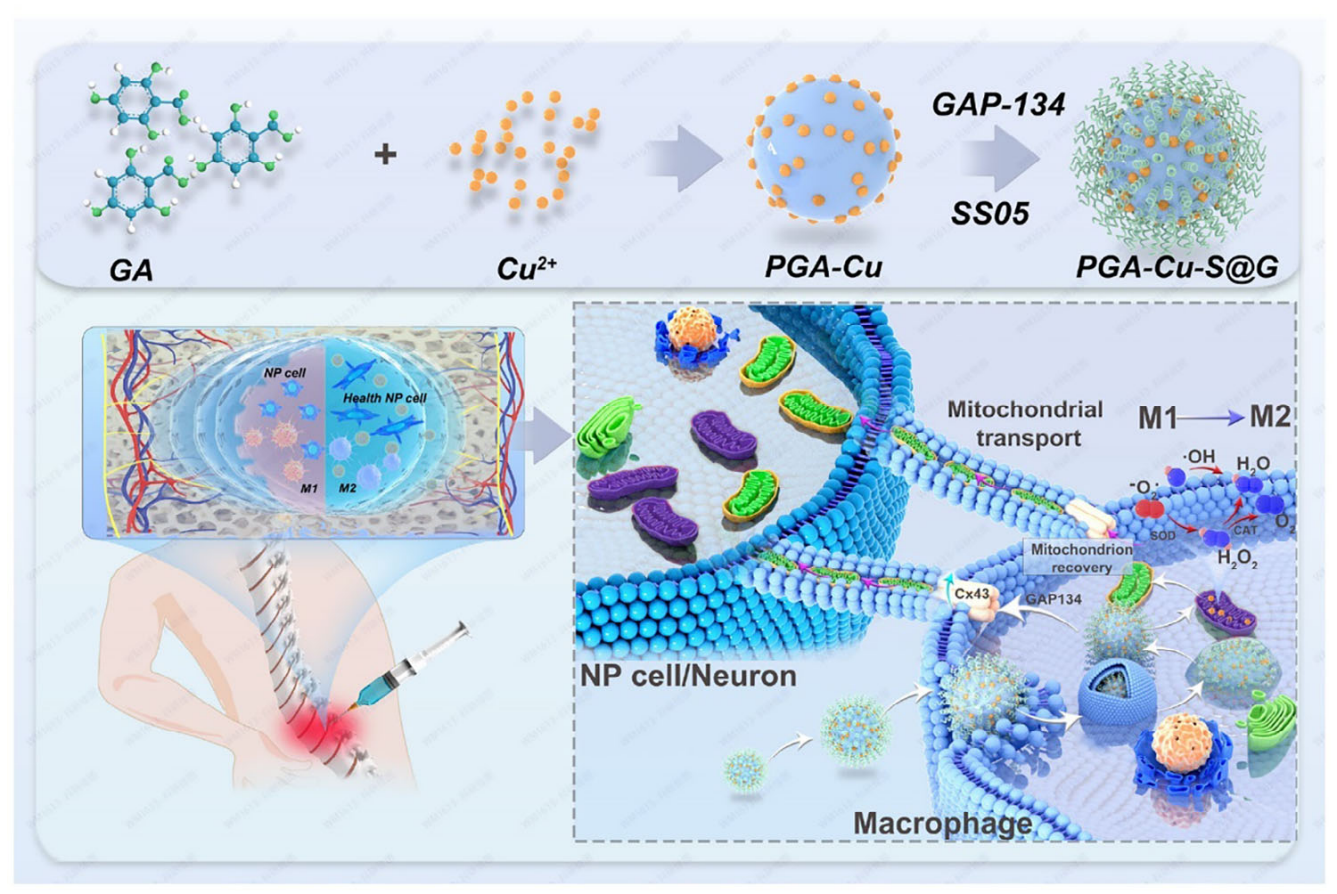
Synergistic Modulating of Mitochondrial Transfer and Immune Microenvironment to Attenuate Discogenic Pain.

This study aims to investigate a therapeutic strategy for IVDD by capitalizing on the potential of macrophage‐mediated mitochondrial transfer. It is hypothesized that the modulation of macrophage polarization to favor M2 macrophages, in conjunction with the enhancement of mitochondrial transfer efficiency, may facilitate the restoration of mitochondrial function in damaged NP cells and neurons. To address the challenges of delivering mitochondria, we propose using nanoparticles to clear excessive ROS in macrophages, thereby improving the quality of donor mitochondria and promoting mitochondrial transfer through gap junction modulation. By focusing on these key mechanisms, we aim to provide insights into a combined immunotherapy and mitochondrial transfer approach that may offer new avenues for treating IVDD (**Scheme** **1**).

## 2 Results

### 2.1 Macrophage Infiltration and Polarization Imbalance Exacerbate IVDD and Discogenic Pain

**Figure 1:**
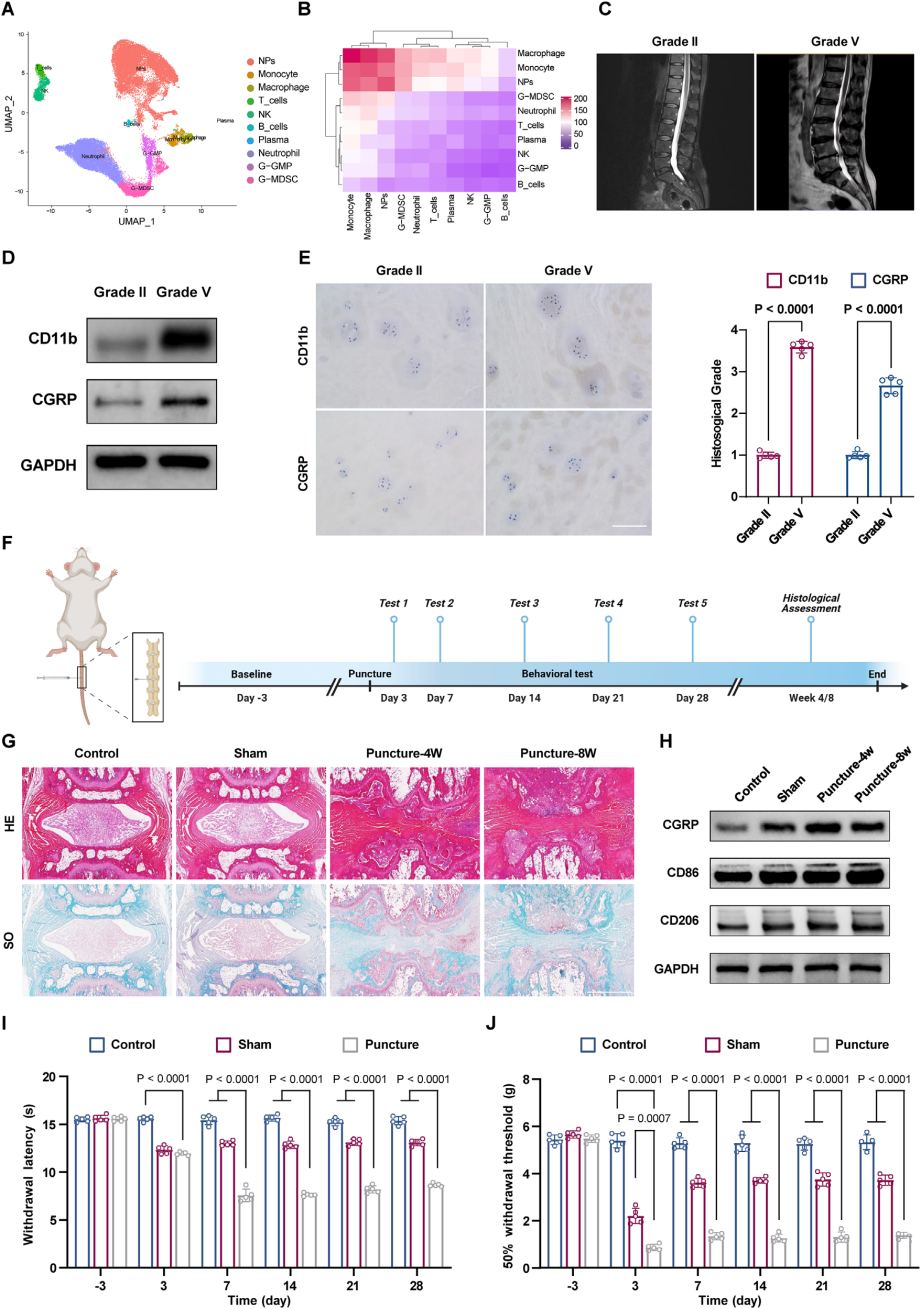
Macrophage infiltration and polarization imbalance exacerbate IVDD and discogenic pain. A) Uniform manifold approximation and projection visualization showing cells inside NP tissues. B) Heatmap showing the number of potential ligand‐receptor pairs between cell groups. C) T2‐weighted magnetic resonance imaging (MRI) was utilized to procure human nucleus pulposus tissues with different degeneration degrees (Grade II and Grade V). D) Western blot analysis of CD11b and CGRP (*n* = 5). E) Immunohistochemical staining was performed to examine the expression of CD11b and CGRP in different degenerating tissues (*n* = 5). Scale bar: 100 µm. F) Experimental design, including establishment of a rat discogenic pain model and subsequent histological and behavioral pain assessments. G) Histological staining images of rat caudal spines (*n* = 5). Scale bar: 1 mm. H) Western blot analysis of CGRP, CD86, and CD206 (*n* = 5). I) Hargreaves tests detecting painful behavior in response to heat stimulation of different groups (*n* = 5). J) Von Frey tests detecting painful behavior in response to mechanical stimulation of different groups (*n* = 5). Data are expressed as mean ± standard deviation.

In order to investigate the potential correlation between macrophage infiltration and IVDD, we employed Uniform Manifold Approximation and Projection for the visualization of immune cells present in the human IVD, utilizing a single‐cell RNA‐seq dataset (GSE165722) from the Gene Expression Omnibus database.^[^ ^20^ ^]^ The results present multiple types of immune cells in degenerated intervertebral discs (**Figure** **1**A). Furthermore, the prediction of interaction networks between different cell types identified macrophages as being involved in numerous interactions with other cell types, particularly with NP cells (Figure 1B). To validate these findings, human nucleus pulposus tissue samples with varying Pfirrmann grades (Grade II and Grade V) were collected and the expression of macrophage marker CD11b and pain neurotransmitter calcitonin gene‐related peptide (CGRP) was assessed (Figure 1C). The results indicated a significant upregulation of CD11b and CGRP expression in Grade V samples in comparison to Grade II samples (Figure 1D and Figure S1, Supporting Information). Furthermore, immunohistochemistry revealed a positive correlation between CD11b and CGRP expression levels and Pfirrmann grades (Figure 1E). These findings indicate that with intervertebral disc degeneration, there is an increase in the infiltration of macrophages and neurons within the IVD.

The findings were additionally validated in the Sprague‐Dawley rat model. A disc puncture at the Co5‐Co6 level was performed in rats to induce IVDD and associated pain behavior (Figure 1F).^[^ ^21^ ^]^ The histological staining of the postoperative specimens demonstrated the successful induction of the discogenic pain model (Figure 1G). Subsequently, protein expression in the IVD was assessed, revealing elevated expression of macrophage markers CD86, CD206, and pain neurotransmitter CGRP in the puncture group relative to the Control and sham groups. It is noteworthy that the M1 macrophage marker CD86 exhibited a notable increase in late‐stage IVDD, whereas changes in the M2 macrophage marker CD206 were not statistically significant in the early and late degeneration stages (Figure 1H and Figure S2, Supporting Information). Furthermore, the pain phenotype was evaluated by administering Hargreaves (thermal pain threshold) and Von Frey (mechanical pain threshold) tests. The findings indicated that on postoperative day 3, the pain threshold slightly declined in the puncture group relative to the sham‐operated group. 1 week following surgery, the puncture group showed significantly reduced pain thresholds compared to the other groups, which persisted for at least 28 d. This suggests that the puncture injury may have induced temperature and mechanical hypersensitivity responses (Figure 1I,J).

It has been demonstrated that activated M1 macrophages secrete proinflammatory cytokines, neurotrophic factors, and matrix metalloproteinases, which exacerbate neuroinflammatory pain and extracellular matrix (ECM) degradation, thereby accelerating IVDD and its associated symptoms.^[^ ^8a^ ^]^ The aforementioned experimental results substantiate the pivotal role of macrophages in the pathogenesis of discogenic pain and underscore their potential as therapeutic targets.

### 2.2 M2 Macrophages Transfer Mitochondria via Nanotubes to NP Cells and Neurons

**Figure 2:**
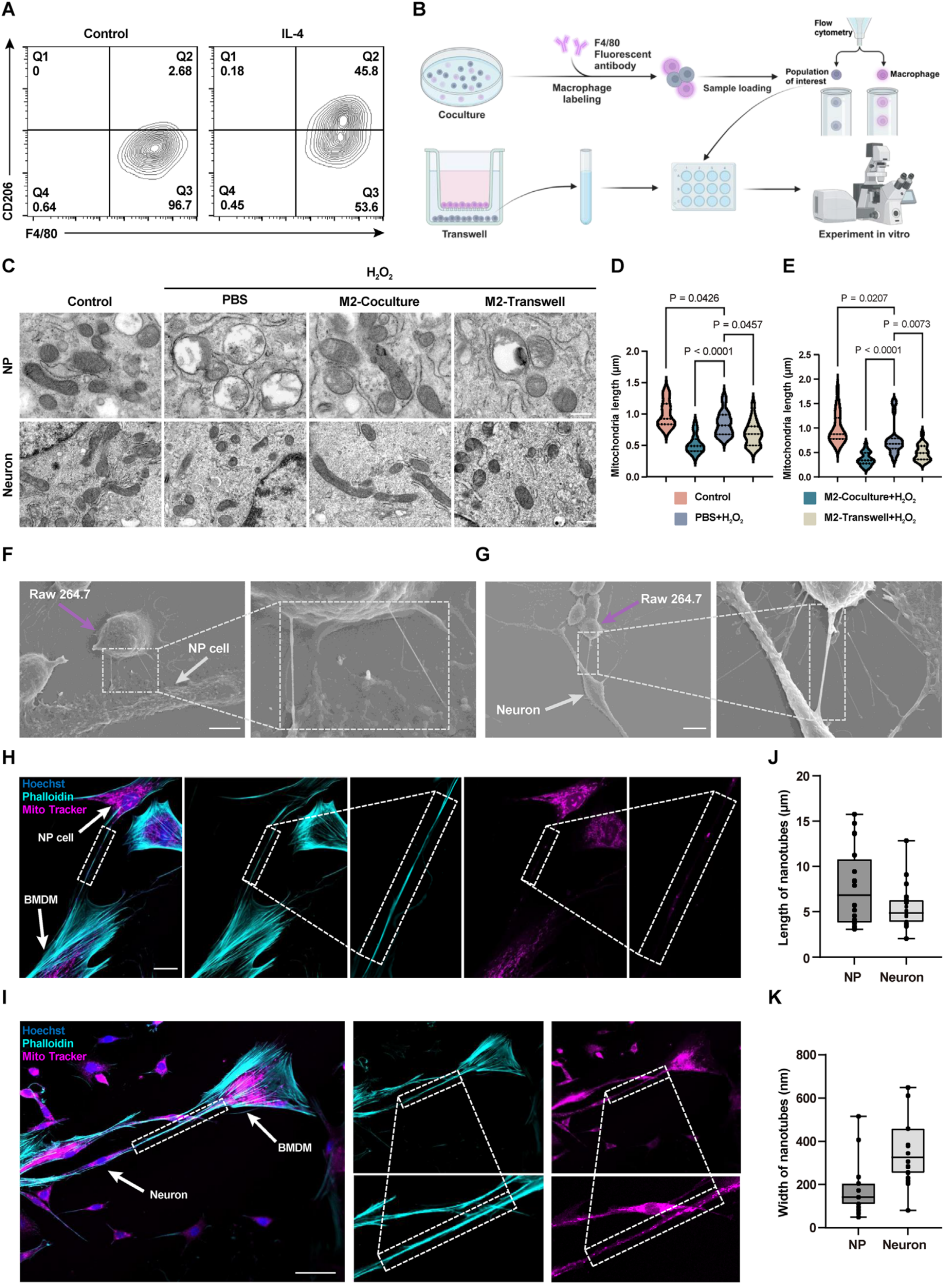
M2 macrophages transfer mitochondria via nanotubes to NP cells and neurons. A) Flow cytometry analysis of F4/80 and CD206 on macrophages. B) Experimental design of two different coculture systems. F4/80‐labeled macrophages separated via fluorescence‐activated cell sorting for subsequent coculture. C) Mitochondrial morphology changes as observed by TEM. Scale bar: 500 nm. D) Quantitative analysis of mitochondrial length in NP cells (*n* = 3). E) Quantitative analysis of mitochondrial length in neurons (*n* = 3). F) SEM images showing nanotubes (white dashed box) between Raw 264.7 (purple arrow) and NP cells (white arrow). Scale bar: 5 µm. G) SEM images showing nanotubes (white dashed box) between Raw 264.7 (purple arrow) and neurons (white arrow). Scale bar: 10 µm. H) Confocal image showing nanotube‐mediated mitochondrial communication between macrophages and NP cells. Scale bar: 25 µm. I) Confocal image showing nanotube‐mediated mitochondrial communication between macrophages and neurons. Scale bar: 50 µm. J) Quantification of nanotube length in SEM images (*n* = 20). K) Quantification of nanotube width in SEM images (*n* = 20). Data are expressed as mean ± standard deviation.

The ratio of M1 to M2 macrophages determines the fate of tissues during inflammatory or injury processes. Anti‐inflammatory M2 macrophages have a high capacity for tissue repair.^[^ ^22^ ^]^ To investigate the therapeutic mechanism of M2 macrophages, we first induced macrophage M2 polarization by interleukin‐4 (**Figure** **2**A). Subsequently, NP cells that had been pre‐exposed to 100 µΜ H₂O₂ were directly cocultured with M2 macrophages. To serve as a control, we established an indirect coculture system utilizing Transwell chambers, which permitted the passage of secreted signaling molecules while preventing direct cell‐to‐cell communication (Figure 2B). Following a 16‐h coculture period, NP cells were isolated using a fluorescence‐activated sorting process and their status examined. The results demonstrated that both coculture systems partially restored the H_2_O_2_‐induced decrease in ATP levels and cell viability. Notably, the coculture group exhibited superior recovery compared to the Transwell group. Similar outcomes were also observed in neurons (Figure S3, Supporting Information).

Mitochondria represent the primary site of cellular ATP production and ROS generation.^[^ ^23^ ^]^ Subsequently, the impact of distinct coculture configurations on mitochondrial functionality was examined. The fluorescence intensity of intracellular ROS was significantly lower in the coculture group than in the Transwell group, as detected by the DCFH‐DA probe (Figure S4, Supporting Information). Furthermore, transmission electron microscopy (TEM) images of mitochondria demonstrated that both coculture models partially restored the H₂O₂‐induced mitochondrial morphological alterations, including swelling, vacuolization, reduction in cristae, and length shortening (Figure 2C). However, NP cells and neurons in the coculture group exhibited a healthier mitochondrial morphology with effective restoration of mitochondrial length compared to the Transwell group (Figure 2D,E).

Based on these experimental results, we hypothesized that there may be cell contact‐dependent mechanisms for M2 macrophages to improve mitochondrial morphology and function in tissue cells. Studies have reported that macrophages can deliver healthy mitochondria to damaged cells via TNTs, vesicles, and other means,^[^ ^14a–c^ ^]^ so we used scanning electron microscopy (SEM) to observe cellular interactions. SEM images revealed nanoscale tubular junctions between macrophages, NP cells, and neurons (Figure 2F,G and Figure S5, Supporting Information). These nanotubes were predominantly 4 to 11 µm long (Figure 2J) and 110 to 450 nm wide (Figure 2K). The observed number of nanotubes may be lower than the actual number because the fragile nanotubes are susceptible to damage during sample preparation and electron microscopy imaging (Figure S6, Supporting Information). Interestingly, nanotubes oriented toward NP cells were slightly longer than those oriented toward neurons, while their widths were reversed. These findings further support the hypothesis that M2 macrophages are involved in cellular communication through direct cell contact. Mito Tracker Red (+) bone marrow‐derived macrophages (BMDM) were cocultured with Mito Tracker Red (−) NP cells or neurons. After 16 h, the cytoskeleton was labeled with ghost pen cyclic peptide and observed under a confocal microscope. Mitochondrial punctate fluorescence was observed in heterogeneous cellular channels composed of the cytoskeleton (Figure 2H,I). Co‐localization of mitochondria with nanotubes confirmed nanotube‐mediated mitochondrial communication between macrophages and NP cells/neurons. These results suggest that mitochondrial transfer between cells under specific pathological conditions is a non‐specific process, mainly influenced by changes in cellular status and environmental conditions, and is not limited by cell type.

### 2.3 Mitochondrial Transfer Regulates the Mitochondrial Function of Recipient Cells

**Figure 3:**
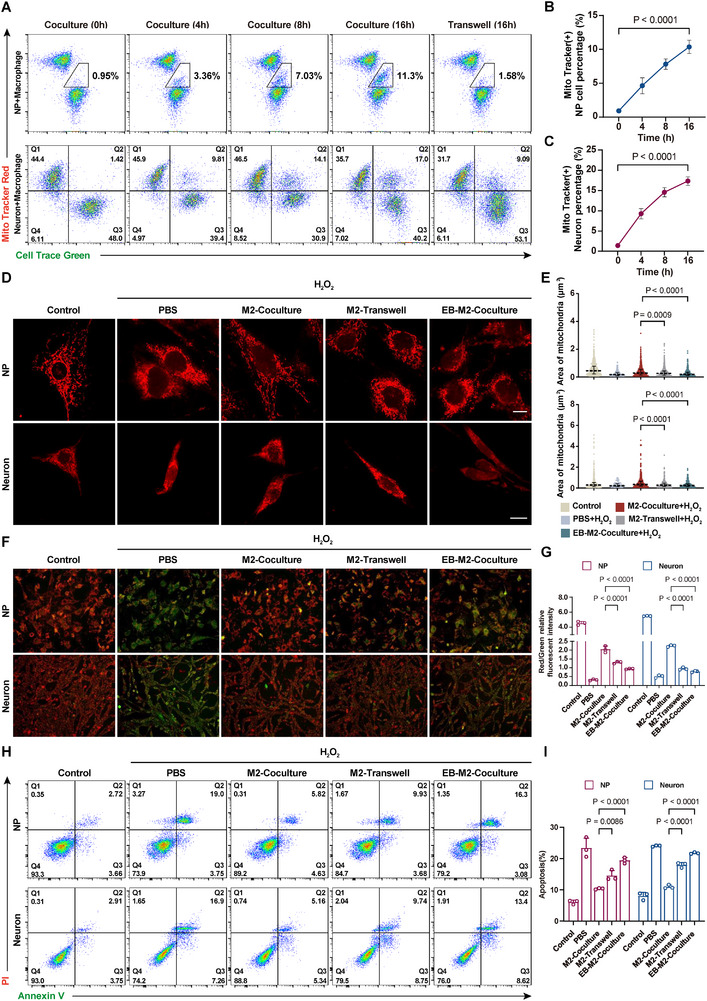
Mitochondrial transfer regulates the mitochondrial function of recipient cells. A) Pseudocolor images showing the proportion of double‐positive cells in different coculture systems at various time points (*n* = 3). B) Quantitative analysis of Mito Tracker (+) NP cell percentage over time (*n* = 3). C) Quantitative analysis of Mito Tracker (+) neuron percentage over time(*n* = 3). D) Representative images of distribution and morphology of mitochondria after different treatments (*n* = 3). Scale bar: 10 µm. E) ImageJ analysis of the area of mitochondria. F) Representative fluorescent images of JC‐1 staining under different treatment conditions (*n* = 3). Scale bar: 100 µm. G) Quantitative analysis of JC‐1 levels under different treatment conditions (*n* = 3). H) Flow cytometry apoptosis analysis of NP cells and neurons under different treatment conditions (*n* = 3). I) Quantitative analysis of apoptosis rate under different treatment conditions (*n* = 3). EB, Ethidium bromide. Data are expressed as mean ± standard deviation.

Using the following staining strategy (Mito Tracker Red to label mitochondria in macrophages, Cell Trance Green to label NP cells and neurons), we quantified the direction and efficiency of mitochondrial transfer under different coculture times and modes by flow cytometry (**Figure** **3**A). Initially, M2 macrophages and NP cells/neurons were labeled in red and green clusters, respectively. Over time, we observed double‐positive cell clusters' appearance and gradual increase, indicating mitochondrial transfer from M2 macrophages to NP cells and neurons (Figure 3B,C). After 16 h of coculture, 11.3% of double‐positive cells were found in NP cells and 17.0% in neurons. The higher transfer efficiency in neurons can be attributed to their abundant synapses, which promote the formation of mitochondrial transfer channels.^[^ ^24^ ^]^ In contrast, no significant change in the percentage of double‐positive cells was observed in Transwell chambers, suggesting that mitochondrial transfer depends on direct cell‐to‐cell contact. To exclude the possibility of non‐specific staining due to dye leakage, we added conditioned media from Mito Tracker Red (+) M2 macrophages and Mito Tracker Red (+) M2 macrophages to parallel cultures of NP cells. The results showed that the addition of M2 macrophages, rather than the addition of conditioned medium, resulted in the appearance of Mito Tracker Red (+) NP cells, demonstrating that the mitochondrial signals in the recipient cells were derived from exogenous mitochondria rather than non‐specific dye leakage (Figure S7, Supporting Information).

To investigate the extent to which different types of macrophages are involved in mitochondrial transfer, we conducted additional flow cytometry experiments to evaluate the mitochondrial transfer capacity of M0, M1, and M2 macrophages. The results demonstrated that M0 and M1 macrophages exhibited minimal or no mitochondrial transfer to damaged NP cells and neurons. In contrast, M2 macrophages displayed a significantly higher level of mitochondrial transfer activity (Figure S8, Supporting Information). This observation suggests that mitochondrial transfer is a unique feature of M2 macrophages, which aligns with their well‐established biological role in tissue repair, regeneration, and anti‐inflammatory responses. The polarization state of macrophages likely determines their mitochondrial transfer capacity, as M2 macrophages are associated with enhanced intercellular communication and metabolic support for damaged cells. By comparison, M1 macrophages, which are more pro‐inflammatory in nature, may prioritize immune defense mechanisms over intercellular material exchange.

Additionally, we performed experiments using the opposite labeling strategy to investigate whether mitochondria could be transferred back from NP cells or neurons to macrophages. Specifically, we pre‐labeled the mitochondria in NP cells or neurons before coculturing them with macrophages. Flow cytometry was then used to detect the presence of exogenous mitochondria in macrophages after coculture. The results showed no significant presence of exogenous mitochondria within macrophages, indicating that NP cells or neurons do not transfer their mitochondria back to macrophages (Figure S9, Supporting Information). These findings suggest that the mitochondrial transfer observed in this study is unidirectional, from macrophages to recipient cells, and does not affect macrophages through reverse transfer.

Subsequently, the impact of mitochondrial transfer on mitochondrial function in recipient cells was investigated. Compared to the Transwell group, the coculture group showed a healthier mitochondrial morphology (Figure 3D). In particular, mitochondrial area and circumference were effectively restored. (Figure 3E and Figure S10, Supporting Information). Excess ROS from oxidative stress disrupts the mitochondrial membrane potential (ΔΨm), leading to mitochondrial depolarization and dysfunction.^[^ ^25^ ^]^ Therefore, we examined the changes in ΔΨm in recipient cells under different treatment conditions. A higher red/green fluorescence ratio represents a normal ΔΨm. Both culture modes partially increased the red/green fluorescence ratio compared to the H_2_O_2_ group, while the coculture group showed a more significant improvement (Figure 3F,G). As the reduction in ΔΨm is an early indicator of apoptosis,^[^ ^26^ ^]^ we investigated the apoptosis rate further. The results showed that coculture reduced the apoptosis rate by 12.3% in H_2_O_2_‐induced NP cells and 13.66% in neurons (Figure 3H,I). However, pre‐disruption of macrophage mitochondria with ethidium bromide (0.5 µM) blocked the recovery of mitochondrial function and apoptosis in recipient cells. This suggests that the additional therapeutic effect mediated by direct contact largely depends on functional mitochondrial delivery rather than other cellular mechanisms associated with direct contact. Thus, improving mitochondrial function in donor cells may contribute to improved recovery of recipient cells.

We also conducted experiments using the microtubule inhibitor Nocodazole, which has been shown in previous studies to suppress the formation of TNTs and thereby inhibit mitochondrial transfer.^[^ ^27^ ^]^ Our SEM images demonstrated that the Nocodazole‐treated group's nanotube connections between macrophages and NP cells or neurons were significantly reduced (Figure S5, Supporting Information). This provides direct evidence that microtubule inhibitors effectively inhibit TNTs formation. Additionally, we evaluated the mitochondrial function of recipient cells. We observed that the addition of Nocodazole not only inhibited mitochondrial transfer but also impaired the ability of M2 macrophages to restore mitochondrial function in damaged cells (Figure S11, Supporting Information). These findings strongly support the conclusion that TNTs are a key pathway for mitochondrial transfer and that the transferred mitochondria are essential in restoring mitochondrial function in recipient cells.

### 2.4 Preparation, Characterization, and ROS Scavenging Capability of PGA‐Cu‐S@G

**Figure 4:**
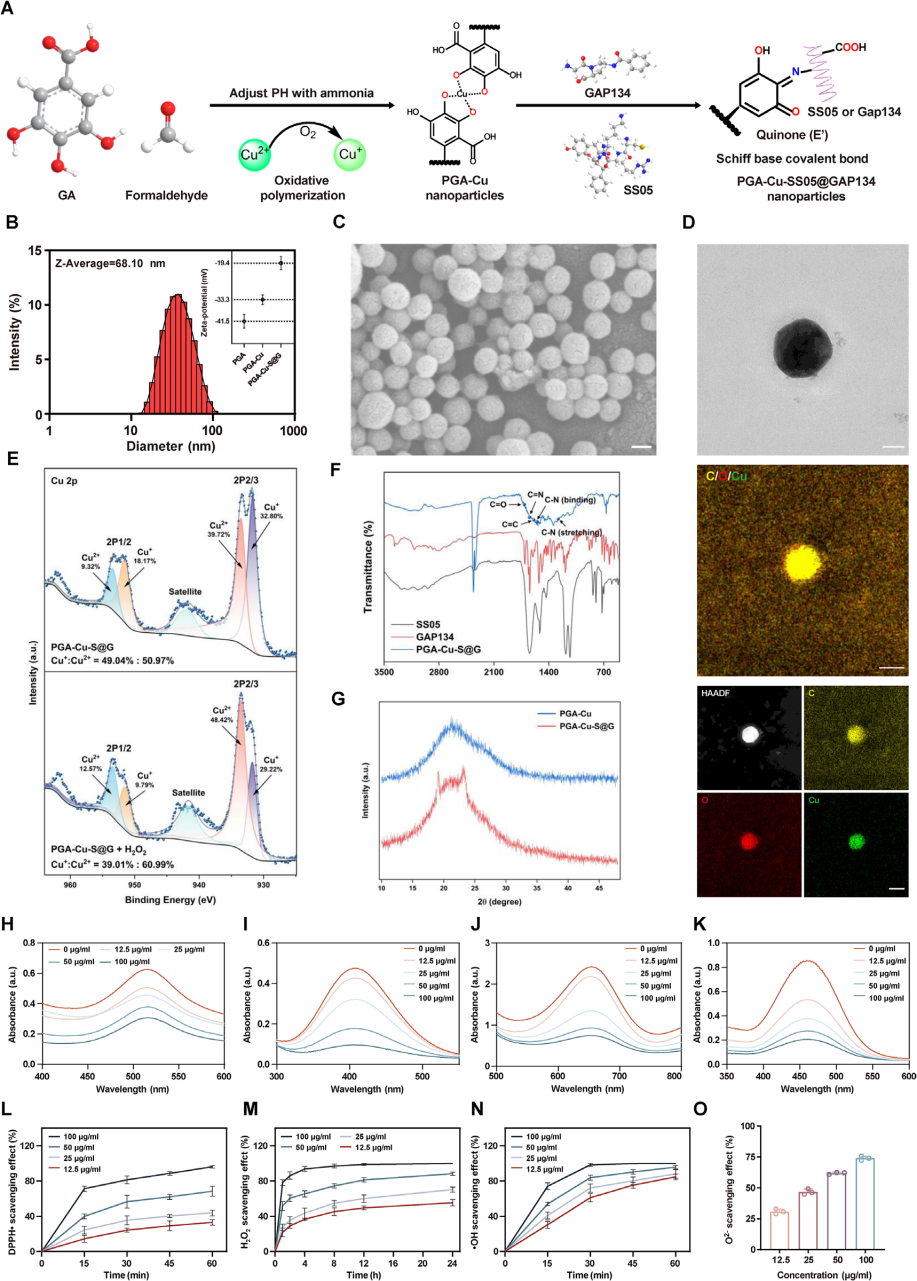
Preparation, characterization, and ROS scavenging capability of PGA‐Cu‐S@G. A) Schematic representation for the synthesis of PGA‐Cu‐S@G. B) Size distribution of PGA‐Cu‐S@G and zeta potentials of different materials. C) SEM image of PGA‐Cu‐S@G. Scale bar: 50 nm. D) TEM (Scale bar: 25 nm) and corresponding energy dispersive X‐ray spectroscopy for PGA‐Cu‐S@G. Scale bar: 50 nm. E) High‐resolution spectra of PGA‐Cu‐S@G for Cu 2p before and after H_2_O_2_ treatment. F) FTIR analysis of characteristic peaks for SS05, GAP134, and PGA‐Cu‐S@G. G) XRD of PGA‐Cu and PGA‐Cu‐S@G. H‐K) UV−vis absorbance spectra showing the radical eliminating activities of PGA‐Cu‐S@G for H) DPPH·, I) H_2_O_2_, J) ·OH, and K) ·O_2_^−^ in 0.5 h (*n* = 3). L) DPPH·, M) H_2_O_2_ and N) ·OH temporal scavenging efficiency of PGA‐Cu‐S@G with various concentrations (*n* = 3). O) ·O_2_^−^ scavenging efficiency of PGA‐Cu‐S@G with various concentrations (*n* = 3). Data are expressed as mean ± standard deviation.

In an alkaline aqueous solution, multiple adjacent phenolic hydroxyl groups of gallic acid (GA) were used to synthesize metal polyphenol nanoparticles (PGA‐Cu) through self‐assembly. Meanwhile, the surface of the nanoparticles contained multiple aldehyde groups that could form Schiff bases with abundant amino groups in two functional peptides (SS05 and GAP134). Finally, PGA‐Cu‐SS05‐GAP134 (PGA‐Cu‐S@G) was formed for further functionalization (**Figure** **4**A). The final binding rate of SS05 is 14.74%, while the binding rate of GAP134 is 15.97% (Figure S12, Supporting Information). PGA‐Cu‐S@G is well dispersed in solution with an average hydrodynamic diameter of about 68.1 nm as measured by dynamic light scattering. The zeta potential of PGA‐Cu‐S@G (−19.4 mV) is significantly higher than that of PGA alone (−41.5 mV) and PGA‐Cu measured at −33.3 mV due to doping with SS05 (2.6 mV) and GAP134 (3.3 mV) (Figure 4B and Figure S13, Supporting Information). SEM and TEM also confirmed that PGA‐Cu‐S@G is a spherical particle with a diameter close to 70 nm (Figure 4C and Figure S14, Supporting Information), composed mainly of carbon (C), oxygen (O), and copper (Cu) elements (Figure 4D), which is attributed to the coordination of Cu^2+^ with the abundant oxygen groups on the PGA surface. X‐ray photoelectron spectroscopy was then performed to investigate the surface composition. The survey spectra confirmed that PGA‐Cu‐S@G is mainly composed of carbon, nitrogen, oxygen, and copper elements (Figure S15, Supporting Information). Gaussian fitting of the high‐resolution Cu 2p spectra showed that copper is present in monovalent (Cu I) and divalent (Cu II) forms, with a ratio of Cu I to Cu II of ≈1:1. However, the intensity of the Cu I peaks decreased. The Cu II peaks increased after H_2_O_2_ treatment. The ratio of the Cu I/Cu II peaks became 2:3. In contrast, the characteristic peaks of the other elements did not change significantly, indicating the importance of the Cu I/Cu II valence transition in the redox process of H_2_O_2_ (Figure 4E). Studies suggest that polyphenolic compounds may enhance the catalytic effect by promoting the valence transition of copper ions,^[^ ^28^ ^]^ thus improving the free radical scavenging efficiency and providing important theoretical support for PGA‐Cu‐S@G as an effective antioxidant.

To quantify the copper content, we performed ICP‐MS analysis on the PGA‐Cu‐S@G samples, which showed that copper accounted for 5.68% of the total mass of the nanoparticles, confirming the effective doping of Cu^2^⁺ (Table S1, Supporting Information). Fourier transform infrared spectroscopy (FTIR) analysis identified the characteristic peaks of SS05 and GAP134, confirming the presence of the main peaks in PGA‐Cu‐S@G (Figure 4F). The characteristic peaks of the C═N stretching vibration appeared in the range of 1620–1650 cm^−1^, indicating that the peptide amino group reacted with the aldehyde group of GA to form Schiff bases, which were effectively incorporated into the nanoparticles. The C═C stretching vibration in poly GA has a characteristic absorption peak at 1590 cm^−1^, and the unreacted carbonyl group has a C═O stretching vibration peak near 1710 cm^−1^. The characteristic peak at 1550 cm^−1^ corresponds to the amide II band of the peptide, and the C‐N stretching vibration peak may appear in the region of 1280 cm^−1^. X‐ray diffraction (XRD) analysis showed that most of the characteristic peaks of GA disappeared in PGA‐Cu‐S@G and PGA‐Cu, probably due to the formation of amorphous structure by oxidative polymerization of GA (Figure 4G and Figure S16, Supporting Information). Moreover, the diameter of PGA‐Cu‐S@G maintained good stability over a long period of time (Figure S17, Supporting Information), providing a reliable basis for its potential biological applications.

Mitochondrial dysfunction is closely associated with sustained high levels of ROS. Therefore, the scavenging of excessive ROS is crucial for PGA‐Cu‐S@G to restore mitochondrial function. To comprehensively investigate the ROS scavenging capacity of PGA‐Cu‐S@G, we selected three major members of the ROS family – hydrogen peroxide (H_2_O_2_), hydroxyl radicals (∙OH), superoxide anions (∙O_2_^−^) and indicators reflecting total antioxidant capacity DPPH∙ and ABTS^+^∙ for detection. The clearance of DPPH∙ was 96.11% after 1 h at 100 µg mL^−1^ concentration (Figure 4H,L); in ABTS^+^∙ analysis, the clearance efficiency was 98.44% after 1 h at 25 µg mL^−1^ concentration (Figure S18, Supporting Information). PGA‐Cu‐S@G showed high sensitivity to ∙OH, with 84.65% clearance after 1 h at 12.5 µg mL^−1^ concentration; at 100 µg mL^−1^ concentration, it was almost completely cleared in 30 min (Figure 4J,N). Furthermore, the results of the SOD‐like enzyme activity assay of PGA‐Cu‐S@G showed that the clearance efficiency of ∙O_2_^−^ was 74.03% at 100 µg mL^−1^ concentration (Figure 4K,O); while for CAT‐like enzyme activity, 93.72% of H_2_O_2_ removal was achieved at 100 µg mL^−1^ concentration for 4 h (Figure 4I,M). These results demonstrate that the coordination polymerization of PGA with copper significantly enhances its antioxidant capacity. PGA‐Cu‐S@G shows excellent in vitro performance in scavenging ROS.

### 2.5 Cellular Internalization, Lysosomal Escape, and Mitochondrial Targeting of PGA‐Cu‐S@G

After confirming the in vitro capabilities of the nanoparticles, we investigated their intracellular properties using cell counting kit‐8 (CCK‐8) to evaluate the toxicity of the nanoparticles on NP cells, neurons, and macrophages. The results showed that peptide modification did not increase the cytotoxicity of PGA‐Cu, and there was no significant effect on cell viability at concentrations of 100 µg mL^−1^ and below. To assess biocompatibility, we cocultured 100 µg mL^−1^ PGA‐Cu‐S@G with the three cell types for 1, 3, and 5 d. Fluorescence images showed that the cell growth rate was not affected. The hemolysis assay also confirmed the safety of PGA‐Cu‐S@G at this concentration (Figure S19, Supporting Information). Therefore, we chose 100 µg mL^−1^ as a safe concentration for further studies.

**Figure 5:**
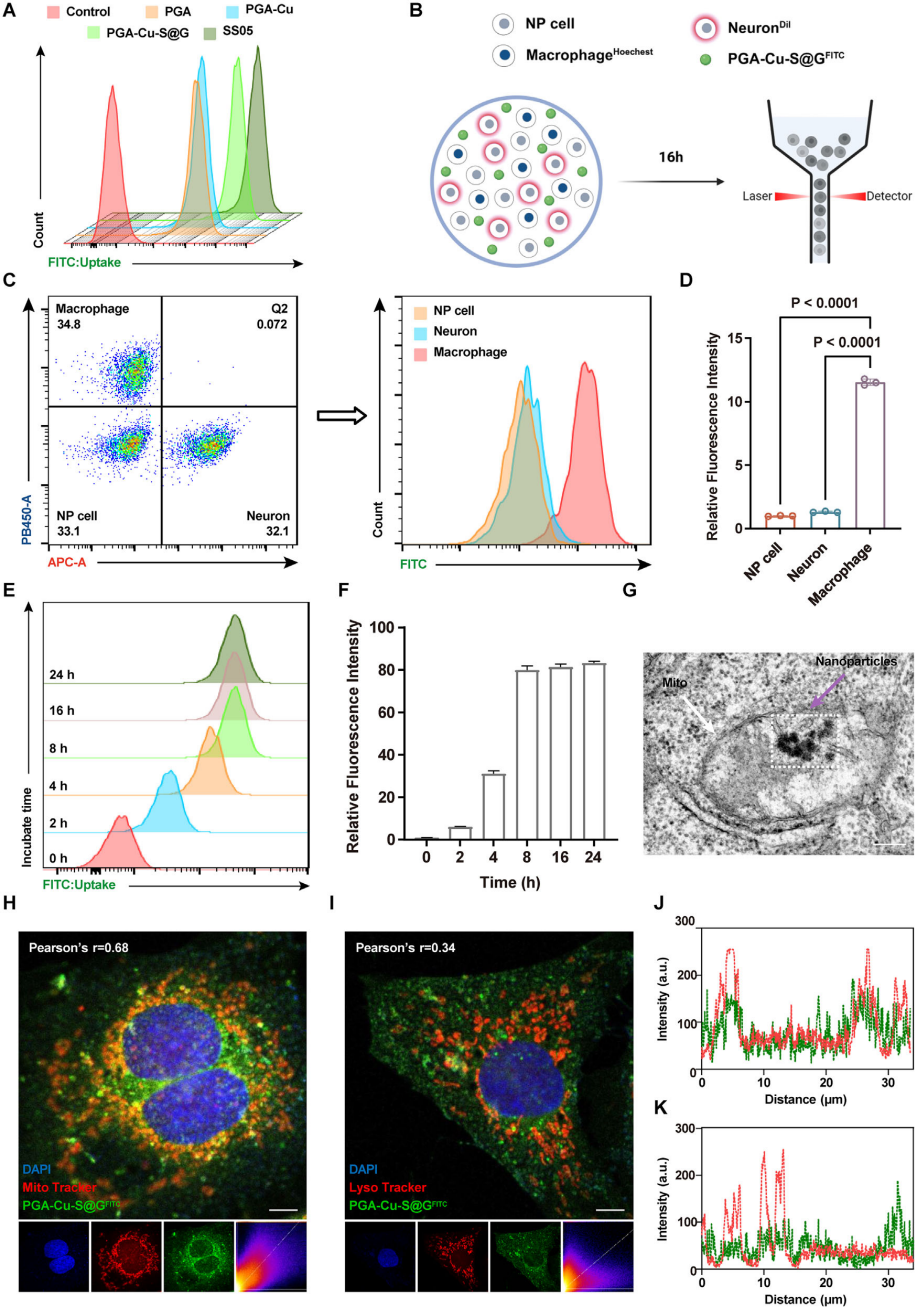
Cellular internalization, lysosomal escape, and mitochondrial targeting of PGA‐Cu‐S@G. A) Evaluation of the binding ability of different materials labeled with FITC to mitochondria (*n* = 3). B) Experimental design of staining strategies for 3 different cell types. C) Gating strategies of 3 different cell types. Hoechst (+) and Dil (‐) cells were defined as macrophages. Hoechst (−) and Dil (+) cells were defined as neurons. Hoechst (−) and Dil (−) cells were defined as NP cells. Flow cytometry was used to measure fluorescence intensity in the FITC channel, reflecting the rate of PGA‐Cu‐S@G internalization by different cells. D) Quantification of FITC mean fluorescence intensity in 3 different cell types (*n* = 3). E) Flow cytometry analysis of PGA‐Cu‐S@G^FITC^ uptake and retention in macrophages over time. F) Quantification of the temporal uptake rate of PGA‐Cu‐S@G^FITC^ by macrophages (*n* = 3). G) TEM image showing the localization of PGA‐Cu‐S@G in macrophage mitochondria. Scale bar: 200 µm. H) Confocal image showing Co‐localization of PGA‐Cu‐S@G^FITC^ with Mito Tracker‐labeled mitochondria. Scale bar: 5 µm. I) Confocal image showing Co‐localization of PGA‐Cu‐S@G^FITC^ with Lyso Tracker‐labeled lysosome. Scale bar: 5 µm. J) Co‐localization of PGA‐Cu‐S@G^FITC^ with Mito Tracker‐labeled mitochondria. K) Co‐localization of PGA‐Cu‐S@G^FITC^ with Lyso Tracker‐labeled lysosome. Data are expressed as mean ± standard deviation.

The affinity of various materials for mitochondria was assessed to reflect their targeting ability. Fluorescein isothiocyanate (FITC)‐labeled materials were co‐incubated with free mitochondria for 1 h at 37 °C. The intensity of FITC fluorescence within the mitochondria was detected by flow cytometry (**Figure** **5**A). The results showed that the mitochondrial affinity of PGA‐Cu‐S@G was second only to that of SS05, which was attributed to the alternating arrangement of aromatic and basic amino acids in SS05, which confers hydrophobicity and lipophilicity. Thus, PGA‐Cu‐S@G effectively targets mitochondria by electrostatic adsorption independent of the ΔΨm. In addition, the surface modification of the peptide made the nanoparticles positively charged surfaces, which increased the affinity of PGA‐Cu‐S@G for negatively charged cells and promoted cellular uptake. We then investigated the differences in the uptake of PGA‐Cu‐S@G by macrophages, NP cells, and neurons. To mimic the pathological microenvironment of the disc, the three cell types were cocultured and distinguished using different staining strategies. After 16 h of co‐incubation with FITC‐labeled PGA‐Cu‐S@G (PGA‐Cu‐S@G^FITC^), cellular uptake rates were quantified by flow cytometry (Figure 5B). The results showed that the intensity of FITC fluorescence within macrophages was more than ten times higher than that of other cells, indicating an absolute dominance of their uptake of PGA‐Cu‐S@G (Figure 5C,D). This may be attributed to the strong phagocytic ability of macrophages.^[^ ^29^ ^]^ As the co‐incubation time increased, the FITC fluorescence intensity in macrophages rose rapidly in the first 8 h and then gradually stabilized (Figure 5E,F).

The subcellular localization of nanoparticles has a significant impact on their therapeutic efficacy.^[^ ^30^ ^]^ Lysosomes present a challenge to the intracellular functioning of nanoparticles.^[^ ^31^ ^]^ It has been demonstrated that nanoparticles with pH‐buffering capacity facilitate the influx of hydrogen ions, chloride ions, and water into the endosome prior to fusion with lysosomes, which results in endosome rupture and prevents lysosomal degradation of nanoparticles.^[^ ^29^, ^32^ ^]^ PGA‐Cu‐S@G exhibits a robust pH‐buffering capacity, which is primarily attributed to its high concentration of carboxylate groups that dissociate within the endosome and enhance the escape ability. To evaluate the lysosomal escape and mitochondrial targeting capabilities of PGA‐Cu‐S@G, we employed a dual‐labeling approach, wherein PGA‐Cu‐S@G was labeled with FITC and mitochondria and lysosomes of BMDM were labeled with Mito Tracker Red and Lyso Tracker Red, respectively. The degree of co‐localization of PGA‐Cu‐S@G with organelles was quantified using Pearson's R‐value. The results demonstrated a notable overlap between the fluorescence signals of PGA‐Cu‐S@G and mitochondria (Pearson's *r* = 0.68) in comparison to lysosomes (Pearson's *r* = 0.34) (Figure 5H,I). Co‐localization quantification maps further demonstrated that the fluorescence of PGA‐Cu‐S@G exhibited a strong correlation with mitochondrial fluorescence, whereas the correlation with lysosomal fluorescence was relatively weak (Figure 5J,K). Additionally, TEM images revealed that PGA‐Cu‐S@G was situated within the mitochondrial matrix of macrophages (Figure 5G). These findings suggest that PGA‐Cu‐S@G can effectively escape from lysosomes and target mitochondria.

### 2.6 PGA‐Cu‐S@G Regulates Macrophage Polarization by Scavenging mtROS

**Figure 6:**
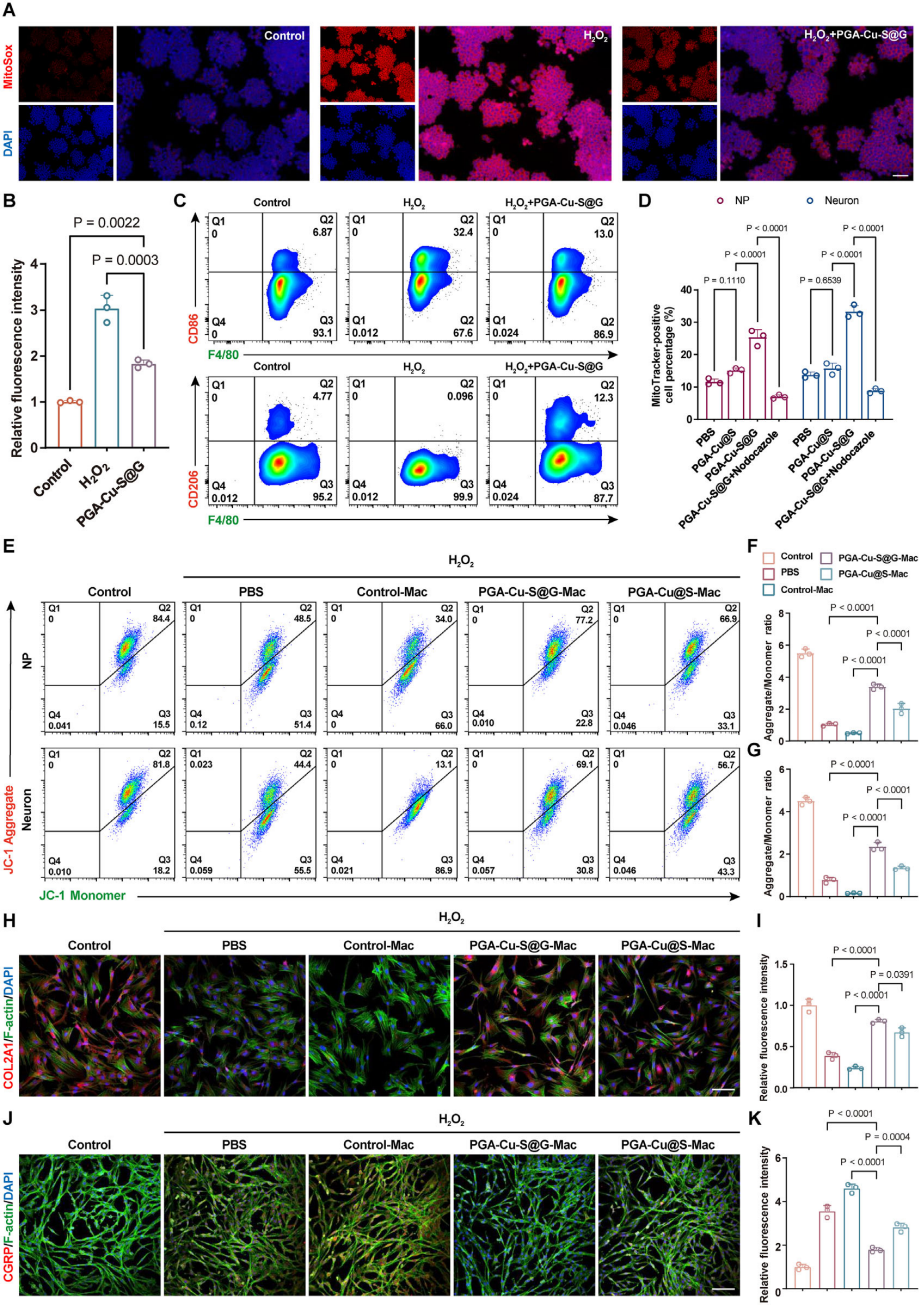
In vitro therapeutic evaluation of PGA‐Cu‐S@G. A) Representative fluorescent images of Mito‐Sox staining under different treatment conditions (*n* = 3). Scale bar: 100 µm. B) Quantitative analysis of Mito‐Sox fluorescence intensity (*n* = 3). C) Flow cytometry analysis of CD86 and CD206 (*n* = 3). D) Quantitative analysis of mitochondrial transfer efficiency (*n* = 3). E) Flow cytometry analysis of JC‐1 levels under different treatment conditions (*n* = 3). F) Quantitative analysis of JC‐1 levels within NP cells under different treatment conditions (*n* = 3). G) Quantitative analysis of JC‐1 levels within neurons under different treatment conditions (*n* = 3). H) Immunofluorescence staining of COL2A1 in NP cells (*n* = 3). Scale bar: 100 µm. I) Quantitative analysis of COL2A1 fluorescence intensity (*n* = 3). J) Immunofluorescence staining of CGRP in neurons (*n* = 3). Scale bar: 100 µm. K) Quantitative analysis of CGRP fluorescence intensity (*n* = 3). Data are expressed as mean ± standard deviation.

The capacity of PGA‐Cu‐S@G to scavenge mtROS to modulate macrophage polarization was subsequently evaluated. To simulate a hypoxic IVDD environment, Raw 264.7 cells were exposed to 100 µM H₂O₂ for 6 h. The effect was then assessed by applying PGA‐Cu‐S@G. MitoSox was used to detect the mtROS level, and the results demonstrated that the mtROS level in the PGA‐Cu‐S@G group was reduced to 60% of that in the H₂O₂ group (**Figure** **6**A,B). Furthermore, flow cytometry showed that PGA‐Cu‐S@G reduced the proportion of H₂O₂–induced MitoSox (+) cells from 40.7% to 23.8% (Figure S20, Supporting Information). These results demonstrated that PGA‐Cu‐S@G effectively scavenges extracellular ROS and mtROS. This capacity was also corroborated in NP cells and neurons (Figure S20, Supporting Information).

The inhibition of ROS generation or the removal of excess ROS has been identified as an effective strategy for modulating macrophage phenotype.^[^ ^33^ ^]^ Accordingly, the impact of PGA‐Cu‐S@G on macrophage polarization was subjected to further examination. Immunofluorescence was conducted to ascertain the mean fluorescence intensity of macrophage polarization markers (CD86 and CD206). The results demonstrated that H₂O₂ treatment resulted in augmented M1 polarization and diminished M2 polarization. In contrast, treatment with PGA‐Cu‐S@G decreased fluorescence intensity for CD86 and increased fluorescence intensity for CD206, which was observed in both Raw 264.7 and BMDM (Figure S21, Supporting Information). Macrophage polarization‐related indexes were detected by flow cytometry (Figure 6C). The data demonstrated that the proportion of F4/80 (+) and CD86 (+) cells in the PGA‐Cu‐S@G group was 13%, which was 19.4% less than that observed in the H₂O₂ group. Conversely, the proportion of F4/80 (+) and CD206 (+) cells (12.3%) was higher than that of the control group (0.096%) and the H₂O₂ group (4.77%). Furthermore, the results of the qRT‐PCR demonstrated that the nanoparticles markedly reduced the expression of M1 macrophage markers (CD80, CD86, iNOS) while concurrently elevating the expression of M2 macrophage markers (CD163, CD206, Arginase) in comparison to the H₂O₂ group (Figure S21, Supporting Information). These findings provide further validation that PGA‐Cu‐S@G has the capacity to modulate macrophage polarization phenotypes, which is attributed to its effective scavenging of mtROS.

### 2.7 PGA‐Cu‐S@G aids M2 Macrophages in Repairing Damaged Cells via Efficient Mitochondrial Transfer

As previously described, TNT is a tubular membrane protrusion that facilitates non‐adjacent cell communication.^[^ ^34^ ^]^ M2 macrophages transfer mitochondria to damaged neurons and NP cells via TNTs. The efficiency of mitochondrial transfer represents a significant limiting factor in the therapeutic efficacy of M2 macrophages. GAP134, a small‐molecule GJC modifier, has been demonstrated to enhance Cx43 expression, thereby promoting GJC formation.^[^ ^35^ ^]^ This, in turn, affects the formation and function of TNTs by modulating the extended terminals that constitute TNTs.^[^ ^19^, ^36^ ^]^ The capacity of PGA‐Cu‐S@G to stimulate Cx43 expression in macrophages was initially investigated. Macrophages were co‐incubated with PGA‐Cu‐S@G and PGA‐Cu@S (PGA‐Cu nanoparticles modified with SS05 only) for 8 h, and the expression of Cx43 was assessed via western blot. The results demonstrated that PGA‐Cu‐S@G elevated the expression of Cx43 in comparison to the control group, whereas PGA‐Cu@S exhibited no notable impact on Cx43 expression (Figure S22, Supporting Information).

Subsequently, the efficiency of macrophage mitochondrial transfer was evaluated through a quantitative flow cytometry assessment. Macrophages that had been pre‐labeled with Mito Tracker Green for mitochondrial labeling were cocultured with NP cells/neurons for 8 h. Macrophages were specifically conjugated with an F4/80 antibody (labeled with APC). The proportion of Mito Tracker Green (+) cells in the APC (‐) cell population was defined as the efficiency of mitochondrial transfer through a gating strategy (Figure S23, Supporting Information). The results demonstrated that the proportion of Mito Tracker Green (+) cells in the PGA‐Cu‐S@G group was markedly higher than that in the other groups. In comparison to the control group, there was a 15% increase in NP cells and an 18.8% increase in neurons, while the addition of nocodazole significantly reduced the proportion of Mito Tracker Green (+) cells (Figure 6D and Figure S23, Supporting Information). These findings suggest that PGA‐Cu‐S@G enhances the efficiency of mitochondrial transfer by promoting TNTs formation through the induction of Cx43 expression.

The subsequent objective was to ascertain whether macrophages treated with PGA‐Cu‐S@G (PGA‐Cu‐S@G‐Mac) demonstrated augmented mitochondrial recuperation (Figure 6E). The results of the ΔΨm analysis indicated that the nanoparticles partially reversed the H_2_O_2_‐induced decrease in ΔΨm. Notably, the Control‐Mac group exhibited the most pronounced ΔΨm depolarization, which may be attributed to the M1‐polarizing tendency of macrophages due to inflammatory factors and ROS secreted by NP cells and neurons under oxidative stress.^[^ ^37^ ^]^ Furthermore, the PGA‐Cu‐S@G‐Mac group showed superior recovery compared to the PGA‐Cu@S‐Mac group, with ΔΨm of NP cells recovered to 61.7% of the control group and ΔΨm of neurons recovered to 52.2% (Figure 6F,G). Subsequently, the expression of functional proteins in NP cells and neurons was assessed. The ECM synthesis protein COL2A1 reflects the regulation of ECM metabolic homeostasis in NP cells, whereas the pain neurotransmitter CGRP reflects the transmission of pain signals in neurons. The results indicated a notable reduction in the fluorescence intensity of COL2A1 in the H_2_O_2_ and Control‐Mac groups, whereas the fluorescence intensity of CGRP exhibited an increase in both groups when compared to the control group. In contrast, both nanoparticles increased COL2A1 expression (Figure 6H,I) and decreased CGRP expression (Figure 6J,K), suggesting an effective alleviation of oxidative stress‐induced ECM remodeling and pain hypersensitivity. NP cells and neurons in the PGA‐Cu‐S@G‐Mac group exhibited the most significant functional recovery. These findings were corroborated by qRT‐PCR results (Figure S24, Supporting Information). The PGA‐Cu‐S@G‐Mac group exhibited the most pronounced restoration of the ECM synthesis function of NP cells and a marked reduction in the synthesis and expression of pain mediators and pain‐related cation channels. The findings indicate that PGA‐Cu‐S@G exerts anti‐inflammatory effects by inducing M2 polarization and facilitating efficient and sustained mitochondrial delivery by macrophages, ultimately improving mitochondrial homeostasis within recipient cells.

### 2.8 In Vivo Therapeutic Evaluation of PGA‐Cu‐S@G

To provide further in vivo evidence for mitochondrial transfer, we conducted an experiment in which we injected Tom20‐GFP‐labelled M2 macrophages derived from rats into the IVD of IVDD rats. After a 48 h incubation period, we extracted the rat IVD and dorsal root ganglion cells for flow cytometry analysis.^[^ ^14^, ^38^ ^]^ For cell identification, we used CD86 and CD24 as markers for NP cells and MAP2 and NeuN as markers for neurons. The results showed that Tom20‐GFP‐positive cells accounted for 16.08% of NP cells and 9.98% of neurons in the gated double‐positive cell population. Notably, when an additional microtubule inhibitor, Nocodazole, was injected, the proportion of Tom20‐GFP‐positive subset in the NP cells and neurons decreased to 9.31% and 4.19%, respectively (Figure S25, Supporting Information). These findings provide direct evidence of mitochondrial transfer from macrophages to damaged cells in vivo and suggest that TNTs play a crucial role in this mitochondrial transfer process.

**Figure 7:**
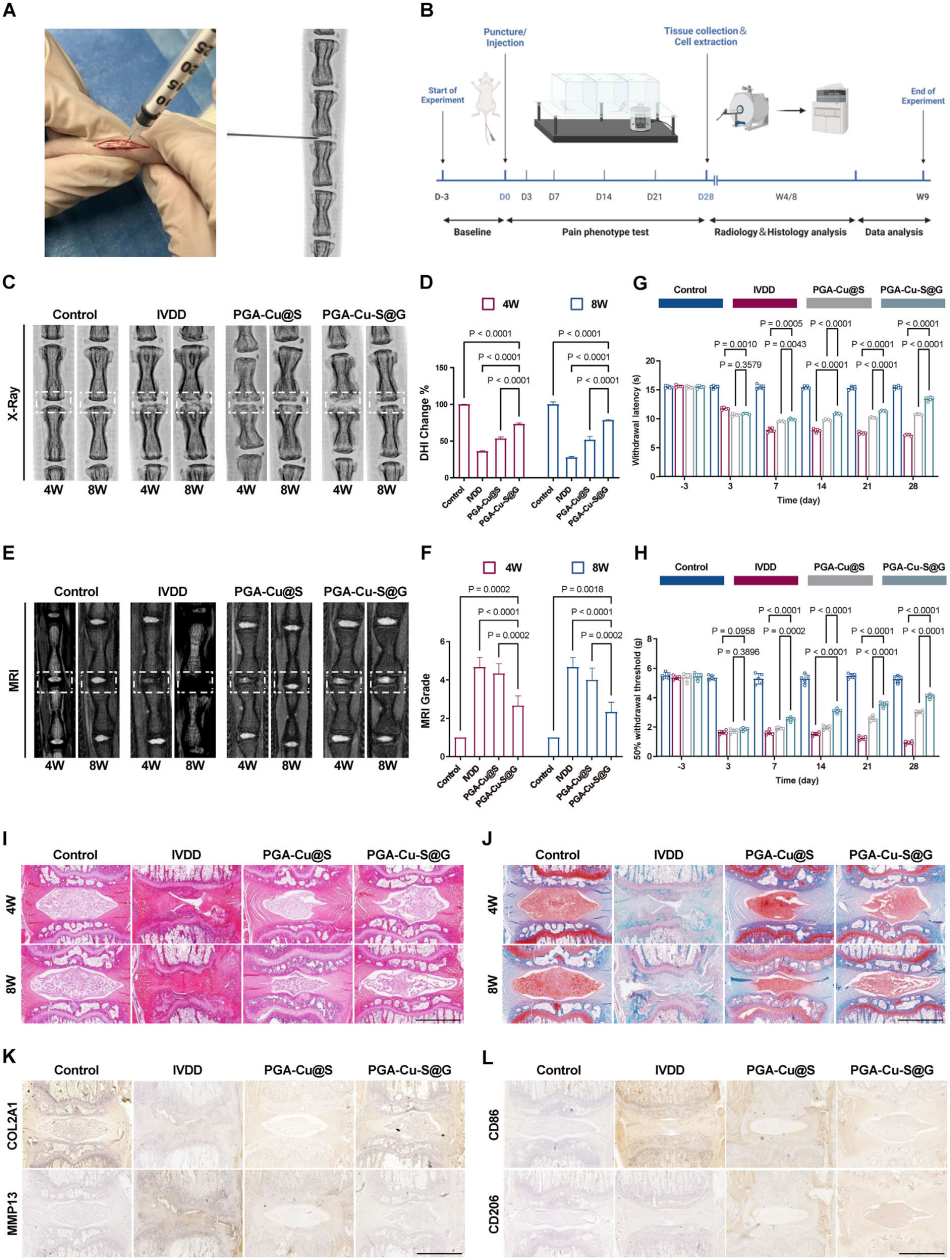
In vivo therapeutic evaluation of PGA‐Cu‐S@G. A) Intraoperative procedures for IVDD. B) Schematic illustration of animal experiments. C) X‐ray images of rat coccygeal vertebrae after different treatments (*n* = 5). D) DHI changes in different groups from 4 to 8 weeks after surgery (*n* = 5). E) MRI images of rat coccygeal vertebrae after different treatments (*n* = 5). F) Pfirrmann grade changes of different groups from 4 to 8 weeks after surgery (*n* = 5). G) Hargreaves tests detecting painful behavior in response to heat stimulation of different groups (*n* = 5). H) Von Frey tests detecting painful behavior in response to mechanical stimulation of different groups (*n* = 5). I) HE staining images of rat caudal spines (*n* = 5). Scale bar: 1 mm. J) SO staining images of rat caudal spines (*n* = 5). Scale bar: 1 mm. K) Immunohistochemistry of COL2A1 and MMP13 at 8 weeks after surgery (*n* = 5). Scale bar: 1 mm. L) Immunohistochemistry of CD86 and CD206 at 8 weeks after surgery (*n* = 5). Scale bar: 1 mm. Data are expressed as mean ± standard deviation.

In parallel, an IVDD model was established in Sprague‐Dawley rats (**Figure** 7A), and different nanoparticles were injected in situ into the diseased segments using a microinjector. The efficacy of the treatment was then assessed radiologically, histologically, and by pain behavior (Figure 7B). In vivo real‐time imaging analysis demonstrated that PGA‐Cu‐S@G was predominantly localized within the IVD and persisted for up to 13 days following a single in situ injection (Figure S26, Supporting Information).

Analysis of the radiographs showed a significant decrease in the disc height index (DHI) in the IVDD group. In addition, significant osteophytes were observed on both sides of the IVD. In contrast, the PGA‐Cu‐S@G group showed the smallest decrease in DHI, which was close to the control group (Figure 7C). MRI is considered the gold standard for the diagnosis of IVDD. According to the Pfirrmann classification, MRI images are graded from grade I to grade V based on signal intensity (Table S2, Supporting Information). The IVDD group showed a significant decrease in disc signal intensity with concomitant bone collapse and fusion. The PGA‐Cu‐S@G group had slightly higher disc signal intensity than the IVDD group but still lower than the PGA‐Cu‐S@G group (Figure 7E). Overall, the changes in DHI and MRI grading were consistent (Figure 7D,F). The results of the pain behavior tests demonstrated that the thermal and mechanical pain thresholds of the IVDD group were significantly lower than the baseline levels and exhibited a declining trend over time. Both nanoparticles demonstrated the capacity to impede the decline in pain threshold. However, the PGA‐Cu‐S@G group showed a more pronounced efficacy in alleviating nociceptive sensitization in comparison to the PGA‐Cu@S group, facilitating the recovery of thermal pain thresholds as early as postoperative day 3 (Figure 7G,H).

The results were further corroborated by histologic analysis. In the tissue bulk map, the morphology of NP tissues in the PGA‐Cu‐S@G group exhibited the greatest similarity to that of the control group (Figure S27, Supporting Information). The cellular structure and morphology of the IVD were observed using hematoxylin and eosin (HE) staining (Figure 7I). Over the course of 4 to 8 weeks, it was observed that the NP cells in the IVDD group underwent gradual replacement by fibroblasts, accompanied by disruption of the boundary between the AF and NP. However, in the PGA‐Cu‐S@G group, the number of NP cells exhibited only slight variation, and the tissue edges remained distinct. The collagen content of the IVDs was evaluated through the use of the Safranin‐O/Fast Green (SO) staining method (Figure 7J). Both nanoparticles demonstrated the capacity to impede the denaturation of the NP. However, the NPs in the PGA‐Cu‐S@G group demonstrated a greater capacity for proteoglycan and collagen enrichment. In conjunction with the histologic grading scale (Table S3, Supporting Information), the PGA‐Cu‐S@G group exhibited the most optimal disc morphology and structural repair (Figure S28, Supporting Information). Immunohistochemistry also demonstrated that PGA‐Cu‐S@G more effectively regulated ECM metabolism (Figure 7K) and reversed macrophage polarization ratio (Figure 7L). Notably, there was no significant difference in immunomodulation between the two types of nanoparticles (Figure S29, Supporting Information). This suggests that the difference in efficacy between the two nanoparticles is not due to the M2 macrophage‐mediated anti‐inflammatory effect but rather the difference in mitochondrial transfer efficiency.

To more accurately simulate the progression and therapeutic effects of IVDD in humans, the Bama pig was selected as a large animal model. The surgical procedure is described in detail in the Methods section. At 8 weeks post‐surgery, MRI revealed that the PGA‐Cu@S group exhibited heightened disc signal intensity relative to the IVDD group, though it was slightly lower than that observed in the PGA‐Cu‐S@G group (Figure S30, Supporting Information). These results indicate that PGA‐Cu‐S@G maintains excellent therapeutic efficacy in a large animal model. Furthermore, HE staining was employed to assess the extent of inflammatory infiltration and tissue damage in vital organs. The findings demonstrated that the nanoparticles exhibited favorable biocompatibility (Figure S31, Supporting Information).

### 2.9 Therapeutic Mechanism of PGA‐Cu‐S@G

**Figure 8:**
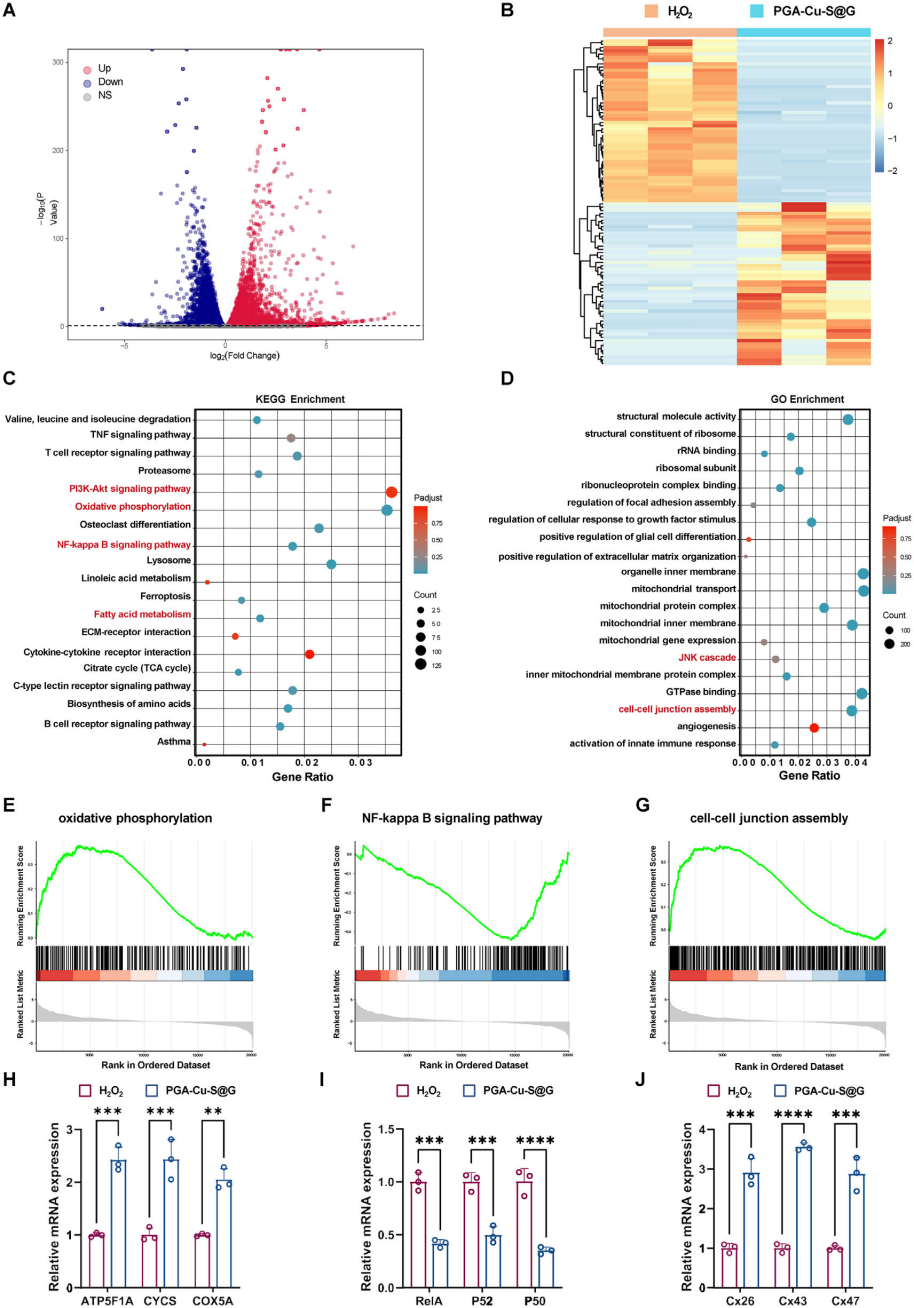
Therapeutic Mechanism of PGA‐Cu‐S@G. A) Volcano plot showing differential gene expression in H_2_O_2_ and PGA‐Cu‐S@G groups. *p* < 0.05, | log2(Fold Change) | ≥ 0. B) Differential gene enrichment maps in H_2_O_2_ and PGA‐Cu‐S@G groups. C) Differential gene pathway enrichment analysis (KEGG analysis). D) GO analysis of differential genes. E–G) GSEA enrichment analysis of oxidative phosphorylation, NF‐𝜅B signaling pathway, and cell‐cell junction assembly between the H_2_O_2_ and PGA‐Cu‐S@G groups. H–J) qRT‐PCR analysis of genes associated with oxidative phosphorylation, NF‐𝜅B signaling pathway, and cell‐cell junction assembly (*n* = 3). Data are expressed as mean ± standard deviation.

The etiology of IVDD is complex and multifactorial, and further elucidation of the specific molecular mechanisms of PGA‐Cu‐S@G in the treatment of discogenic pain is necessary. Transcriptomic sequencing analysis was conducted on macrophages treated with PGA‐Cu‐S@G. The treatment resulted in the up‐regulation of 3763 genes and the down‐regulation of 3806 genes in comparison to the H_2_O_2_ group (**Figure** **8**A). The data were subjected to heat map analysis for visualization (Figure 8B). H_2_O_2_ induces cellular oxidative stress and activates various stress‐related signaling pathways, leading to significant changes in gene expression. PGA‐Cu‐S@G treatment reversed most of the changes in gene expression induced by hydrogen peroxide, suggesting that the nanoparticles may be useful in alleviating oxidative stress. The Kyoto Encyclopedia of Genes and Genomes (KEGG) enrichment analysis indicated that the differentially expressed genes (DEGs) were predominantly associated with metabolic and inflammatory pathways. With regard to metabolic processes, notable discrepancies were discerned in oxidative phosphorylation, Tricarboxylic acid cycling, and fatty acid metabolism between the H_2_O_2_ and PGA‐Cu‐S@G groups. This suggests that nanoparticles may regulate macrophage phenotypes by modulating these processes. With regard to the inflammatory response, the inhibition of the NF‐𝜅B signaling pathway and the up‐regulation of the PI3K‐Akt signaling pathway were of particular note. These changes may be related to the increase in the number of M2 macrophages induced by PGA‐Cu‐S@G (Figure 8C). Gene ontology (GO) analysis revealed that the DEGs between the H_2_O_2_ and PGA‐Cu‐S@G groups were primarily associated with ribosomes and mitochondria. These organelles play a pivotal role in regulating oxidative stress and protein synthesis, respectively. Nanoparticles affected the immune phenotype and metabolic activity of macrophages by modulating the function of these two organelles (Figure 8D). Furthermore, the nanoparticle group exhibited a notable increase in the function of cell‐cell junction assembly, which may be associated with GAP134‐mediated promotion of Cx43 expression or the nanoparticle‐induced expression of Cx43 through the JNK pathway.^[^ ^16^, ^18^ ^]^

The gene set enrichment analysis (GSEA) demonstrated that PGA‐Cu‐S@G was capable of upregulating oxidative phosphorylation (Figure 8E) and downregulating the NF‐𝜅B signaling pathway (Figure 8F). Furthermore, PGA‐Cu‐S@G was observed to enhance the function of intercellular junction assembly, thereby demonstrating the potential to promote mitochondrial transfer (Figure 8G). The results of the GSEA analysis were further validated by qRT‐PCR, which demonstrated that the expression of genes associated with oxidative phosphorylation and intercellular junction assembly was increased (Figure 8H,J), while the expression of genes linked to the NF‐𝜅B signaling pathway was decreased following treatment with PGA‐Cu‐S@G (Figure 8I). These findings elucidate the underlying mechanisms of this smart nanoparticle system to alleviate discogenic pain, demonstrating its potential as an innovative strategy for clinical translation.

## 3 Conclusion

In conclusion, this study reveals the use of nanoparticles to coordinate the synergistic regulation of the immune microenvironment and mitochondrial transfer, thereby providing an effective approach to the treatment of discogenic pain. The findings demonstrated that PGA‐Cu‐S@G nanoparticles with effective mitochondrial targeting significantly augmented mitochondrial mass and transfer efficiency in macrophages, thereby ensuring mitochondria's effective and sustained delivery to damaged cells. This offers a solution to the efficiency and quality challenges of mitochondrial delivery. Furthermore, the synergistic application of immunotherapy and mitochondrial transfer therapy establishes a foundation for the treatment of IVDD, thus advancing the practical clinical application of this therapeutic approach.

## 4 Experimental Section

### Ethics Approval

Ethics involved in experimental arrangement and implementation in this study, including patient medical data consultation, NP tissue sample collection and application, animal obtainment, animal surgical operation, and animal sample collection, were approved by Medical Ethics Committee of the Second Affiliated Hospital of Wenzhou Medical University (No. LCKY2020‐157) and Laboratory Animal Ethics Committee of Wenzhou Institute, University of Chinese Academy of Sciences (No. WIUCAS23120101, No. WIUCAS24040303).

### Single‐Cell Transcriptome Sequencing Data Acquisition and Analysis

Single‐cell RNA‐seq data from discs with different degrees of degeneration were self‐loaded from the Gene Expression Omnibus data repository (GSE165722). The Seurat package was used for cell normalization and regression based on the expression table according to the percentage of mitochondria rate to obtain the scaled data. To correct the batch effect, the RunHarmony function from the Harmony package was applied. PCA was constructed based on the scaled data with the top 2000 highly variable genes, and the top 40 principals were used for tSNE construction and Uniform Manifold Approximation and Projection construction. The marker genes of each cluster were determined as described above. Cell communication analysis was performed using the CellChat package.

### Human NP Tissue Sample Collection

NP tissue samples from patients with lumbar intervertebral disc herniation who underwent discectomy were evaluated using the Pfirrmann grading system, which classified the degenerated IVD tissue as Grade II and Grade V. Additionally, these patients, who did not have any cardio‐cerebral‐vascular disease, cancers, infection, immune and endocrine diseases, or organ dysfunction, approved the use of NP tissue samples in scientific studies. All volunteers provided informed consent for using their NP tissues in medical research. The study population consisted of patients aged 35–65 (*n* = 5).

### Animal Model

The Sprague‐Dawley rat was chosen as the experimental animal for this study. All rats were sourced from the Zhejiang Provincial Laboratory Animal Center (Hangzhou, China). To establish the discogenic pain model, the sample size was *n* = 5 for each of the three groups: 1) no surgical incision (control), 2) a skin incision without injury to the disc tissues (sham), and 3) Co5‐Co6 NP injury (puncture). To validate the therapeutic effects of the nanoparticles in vivo, sample sizes were *n* = 5 for each of the four groups (Control, IVDD, PGA‐Cu@S, and PGA‐Cu‐S@G). In particular, following the administration of 2% (w/v) pentobarbital (40 mg kg^−1^), rats weighing between 250 and 300 grams were selected for needle puncture surgery. A 22G needle was utilized. The punctures were performed at the fifth/sixth caudal vertebrae (Co5‐Co6) with the assistance of radiographic guidance. A needle was inserted into the intervertebral disc space at a fixed depth of 3 mm. Following insertion, the needle was rotated 360° and left in place for 30 s. Thereafter, 10 µL of the material (PBS; PGA‐Cu@S (100 µg mL^−1^); PGA‐Cu‐S@G (100 µg mL^−1^)) was injected into the IVD with a micro syringe. The injections were administered weekly. Mechanical and thermal pain thresholds were assessed at various time points. Tissue degeneration levels were evaluated using the DHI method and graded according to the Pfirrmann grading system with the assistance of radiography and MRI. The grading was reviewed by three experts in the field of orthopedics.

Bama pigs (*n* = 3, aged 12 months, skeletally mature, weight 20–30 kg) underwent partial nucleotomy under general anesthesia with isoflurane. The five lumbar vertebrae (L1‐L5) were exposed, and partial nucleotomy was performed on L2‐L3, L3‐L4, and L4‐L5 with the 16G needle. Subsequently, 0.5 mL of the phosphate buffered saline (PBS), PGA‐Cu@S (100 µg mL^−1^), and PGA‐Cu‐S@G (100 µg mL^−1^) were administered into the L2‐L3, L3‐L4, and L4‐L5 discs, respectively. The L1‐L2 disc, which did not undergo nucleotomy, was used as a control. The incision was then closed using 0‐0 nylon sutures.

### Macrophage Isolation and In Vitro Differentiation

To obtain bone marrow‐derived monocytes, the tibia and femur bones were flushed with PBS. To generate monocyte‐derived macrophages, 10⁶ cells were seeded in a 75 cm^2^ non‐treated tissue culture flask (NEST, Jiangsu, China) for 7 days in high‐glucose Dulbecco's Modified Eagle medium (DMEM; Gibco 11 965 092) and DMEM/F12 medium (Gibco 12 634 028) (1:1). The medium was supplemented with 30% L929 cell‐conditioned medium, 10% fetal bovine serum (FBS; Gibco A5670701), 1% penicillin‐streptomycin (Gibco 15 140 122), and 1% L‐glutamine (200 mM, Solarbio G0200). To polarize the macrophages toward M2 macrophages, the cells were stimulated with 20 ng mL^−1^ of IL‐4 (Solarbio P00196) for 24 h.

### Cell Culture

NP cells — NP tissues were isolated from Sprague‐Dawley rats and incubated in 2 mg mL^−1^ collagenase II at 37 °C for 2 h. After centrifugation to remove the collagenase, NP cells were cultured in DMEM/F12 medium (Gibco 12 634 028) supplemented with 10% FBS (Gibco A5670701) and 1% penicillin‐streptomycin (Gibco 15 140 122).

PC‐12 cells — PC‐12 cells (ATCC, CRL‐1721) were seeded at a density of 1 × 10^4^ to 5 × 10^4^ cells mL^−1^ in RPMI‐1640 medium (Gibco 11 875 093) containing 10% FBS (Gibco A5670701) and 1% penicillin‐streptomycin (Gibco 15 140 122).

Raw 264.7 cells — The Raw 264.7 cells (ATCC, TIB‐71) pellet was resuspended in fresh pre‐warmed complete high‐glucose DMEM medium (Gibco 11 965 092) with 10% FBS (Gibco A5670701) and 1% penicillin‐streptomycin (Gibco 15 140 122). Cells were then seeded into a culture flask at a density of ≈1 × 10^5^ to 2 × 10^5^ cells mL^−1^.

All cells were maintained in a 37 °C incubator with 5% CO_2_, and the culture medium was refreshed every 2 days.

### Isolation of Cells from the Coculture System

NP cells and PC‐12 cells were isolated using a fluorescence‐activated sorting cytometer (Beckman, Brea, USA). In brief, the cocultured cells were harvested through trypsinization and suspended in neutral PBS. Initially, the mixed cell population was gated, followed by the selection and collection of F4/80‐negative cells. A schematic representation of the fluorescence‐activated sorting cytometer process is shown in Figure 2B.

### Cell Viability Assessment

Cell viability was evaluated using a CCK‐8 assay (Beyotime, Shanghai, China). Cells were plated at a density of 5 × 10^3^ cells per well in 96‐well plates (NEST, Jiangsu, China). A working solution was then prepared by mixing the CCK‐8 solution with a serum‐free medium in a 1:9 ratio, following the manufacturer's instructions. This solution was incubated with the cells in the dark at 37 °C for 2 h, after which absorbance was measured at 450 nm. On days 1, 3, and 5, the cells were treated with 250 µL of Calcein‐AM/propidium iodide detection solution (Beyotime, Shanghai, China) for 30 min and then examined under a fluorescence microscope. Live cells exhibited green fluorescence, while those with damaged membranes appeared red.

### Measurement of Intracellular ATP Levels

Intracellular ATP levels were determined using an ATP assay kit (Beyotime, Shanghai, China) following the manufacturer's guidelines. Adherent cells or tissue samples were lysed with lysis buffer and centrifuged at 12 000 × g for 5 min at 4 °C. The resulting supernatants were collected for a photochemical reaction with ATP working solutions. A luminometer was used to measure the light units from both the samples and ATP standard solutions. ATP levels were then calculated based on a calibration curve derived from the ATP standards.

### Mitochondrial Analysis

Cells were collected using trypsin and fixed with an electron microscope fixative (Servicebio G1102). They were then treated with 0.1 M phosphate buffer containing 1% osmium tetroxide (Ted Pella Inc. 18 456) for fixation. Following this, the cells were dehydrated through a graded ethanol series and embedded. Ultrathin sections, ≈60−80 nm thick, were produced using an ultrathin sectioning machine. The sections were poststained with dicumyl acetate and lead citrate for transmission electron microscopy analysis (HITACHI HT7700, Japan).

Cells were seeded in confocal dishes and co‐incubated with Mito Tracker Red (Thermo Fisher Scientific) for 30 min. After this, the cells were observed and imaged with a confocal microscope. JC‐1 (Beyotime, Shanghai, China) was applied for 30 min before imaging to assess the membrane potential of live cells. Superoxide production was measured using MitoSox reagent (40778ES50; Yeasen Biotechnology, Shanghai, China), which was initially dissolved in dimethyl sulfoxide to create a 5 mM stock solution, then diluted to a 5 µM working solution. This working solution was added to a 12‐well plate with the cells. All cell nuclei were stained with DAPI (Thermo Fisher Scientific) and visualized using a laser confocal microscope (Olympus).

### Scanning Electron Microscopy

Cells were cultured on 12‐mm‐diameter glass coverslips for the required duration as specified by the experiment. The samples were fixed using 2.5% glutaraldehyde (Sigma) in 0.1 M sodium cacodylate buffer (Electron Microscopy Sciences). After fixation, the cells were washed three times for 15 min each with 0.1 M sodium cacodylate buffer, then post‐fixed in 0.1% OsO_4_ (Sigma) in water for 1 h at room temperature, followed by two washes of 10 min each with water before dehydration. The dehydration process included 5 min in 35% ethanol, 5 min in 50% ethanol, 10 min in 70% ethanol, 10 min in 90% ethanol, and two rounds of 100% ethanol for 10 min each. Once fixed and dehydrated, the coverslips were dried and placed on SEM stubs for sputter coating using an EMS 300T D dual‐head sputter coater with gold or platinum/palladium (5 nm). Imaging was conducted with a Zeiss FESEM Supra 55‐VP microscope, and the images were processed with ImageJ software.

### Synthesis of Nanomaterials

Step 1: dissolve 0.2 g of F127 in a mixed solvent of water (50 mL) and ethanol (25 mL). Step 2: add 0.1 g of gallic acid to the solution and stir until fully dissolved. Then, add 0.25 mL of ammonia solution (25% w/w) and stir mildly at room temperature for 30 min. Step 3: add 50 µL of formaldehyde solution and continue the reaction for 30 min. Step 4: add 1 mL of a 10 mg mL^−1^ CuCl_2_ solution (37% w/w) to the mixture and stir for 1 h. Step 5: transfer the mixture to a high‐pressure reactor (100 mL) and perform hydrothermal treatment at 100 °C for 8 h. Next, the solution was centrifugated at high speed and washed thrice with distilled water and ethanol. The solution was vacuum‐dried and dried to obtain PGA‐Cu powder. Subsequently, PGA‐Cu and SS05/GAP134 were dissolved in distilled water at a mass ratio of 25:1 and stirred at a constant speed for 6 h. The uncoupled peptides were removed by washing three times with distilled water and ethanol. Finally, the solution was vacuum‐dried and dried to obtain PGA‐Cu‐S@G powder. To determine the final binding rate of SS05 and GAP134 with PGA‐Cu, the characteristic absorption peak at 275 nm and 223 nm was measured using a UV spectrophotometer. As previously described, the peptide (n_1_) was combined with PGA‐Cu of mass N. Subsequently, the mixture underwent washing and centrifugation using absolute alcohol. The resulting supernatant was collected, and the unbound SS05 (n_2_) mass was calculated by measuring the absorbance at 275 nm in the supernatant. Similarly, the mass of unbound GAP134 was calculated by measuring the absorbance at 223 nm in the supernatant.

(1) $$\mathrm{Binding}\;\mathrm{rate}\left(\%\right)=\frac{n_{1}-n_{2}}{N}\times 100\%$$

(2) $$\mathrm{SS}05\;\mathrm{Binding}\;\mathrm{rate}\left(\%\right)=\frac{4000-2526}{100000}\times 100\%=14.74\%$$

(3) $$\mathrm{GAP}134\;\mathrm{Binding}\;\mathrm{rate}\left(\%\right)=\frac{4000-2403}{100000}\times 100\%=15.97\%$$

### In Vitro Mitochondrial Targeting Validation

Following the instructions of the mitochondrial isolation kit, macrophages were collected and mechanically homogenized. The mitochondria were then isolated through centrifugation. After isolation, the mitochondria were co‐incubated with FITC‐labeled materials for 1 h at 37 °C. The mitochondria were subjected to three washes in PBS to remove any residual unbound materials. Finally, the fluorescence of the mitochondrial suspension was assessed using a flow cytometer.

### Fluorescence Microscopy

Cells cultured on 12‐mm‐diameter coverslips were fixed with 4% paraformaldehyde at room temperature for 2 h. After fixing, the cells were washed thrice with 1 × PBS for 20 min each. They were then permeabilized by incubating with 0.5% Triton X‐100 at 4 °C for 10 min, followed by three washes with 1 × PBS. The cells were blocked with a 10% bovine serum albumin solution at room temperature for 1 h, and then incubated with the primary antibody overnight at 4 °C. This was followed by staining with a secondary antibody (1:300 dilution) for 1 h at room temperature. For actin staining, the cells were treated with 50 µg/ml of Alexa Fluor 488 phalloidin (Thermo Fisher Scientific) for 1 h at room temperature. Mitochondria were stained with Mito Tracker Red (Thermo Fisher Scientific) for 30 min, and the nuclear were stained with DAPI (Thermo Fisher Scientific) for 15 min. The cells were washed three additional times with 1 × PBS. Coverslips were then mounted onto glass slides, and images were captured using a Nikon Eclipse Ti camera (Nikon Instruments) with NIS‐Elements imaging software. ImageJ software was used for post‐processing. The antibodies utilized for immunostaining included CD86 (#91882T; CST, USA), CD206 (#24595T; CST, USA), COL2A1 (ab307674; Abcam, UK), and CGRP (ab272713; Abcam, UK).

### Cell Apoptosis and Necrosis

Cells were collected following the manufacturer's instructions and gently pipetted into a single‐cell suspension using 100 µL of 1 × Binding Buffer. Next, 5 µL of Annexin V‐FITC and 5 µL of PI Staining Solution (Beyotime, Shanghai, China) were added. The cells were incubated in the dark at room temperature for 10 min, after which 400 µL of 1 × Binding Buffer was added and gently mixed. Finally, the apoptosis and necrosis rates of the cells were analyzed using a CytoFLEX (Beckman Coulter, USA).

### Western Blotting

Cellular proteins were extracted by lysing the cells in a RIPA lysis and extraction buffer containing protease and phosphatase inhibitors (Thermo Fisher Scientific). Protein concentrations were then normalized using a BCA protein assay kit (Beyotime, Shanghai, China). Then, electrophoresis separated proteins on 8–12% sodium dodecyl sulfate‐polyacrylamide gels and transferred to polyvinylidene fluoride membranes. The membranes were sealed with a rapid‐closure solution (Beyotime, Shanghai, China) at room temperature for 20 min before being incubated overnight at 4 °C with the primary antibody. After that, the membranes were incubated for 1 h at room temperature with the appropriate secondary antibody, and detection was performed using an Enhanced ECL Chemiluminescence Detection Kit (Vazyme, Nanjing, China). Detailed information about the antibodies used can be found in Table S4, Supporting Information.

### Quantitative Real‐Time PCR

Cells were grown in 6‐well plates, and after different treatments, total RNA was extracted using a Total RNA Extraction Kit (Beyotime, Shanghai, China). According to the manufacturer's instructions, cDNA was synthesized using a Prime Script RT kit (Takara, Kyoto, Japan). An RT‐PCR system (LightCycler 96; Roche, Basel, Switzerland) was used for amplification. The RT‐PCR parameters were as follows: 40 cycles of 30 s at 95 °C, 5s at 95 °C, and 30 s at 60 °C. Primer sequences are available in the Table S5, Supporting Information.

### X‐Ray and MRI Examinations

X‐ray images of rat tails were taken before surgery and at 4 and 8 weeks post‐surgery using an XPERT.8 X‐ray machine (Kubtec, Stratford, CT, USA). The DHI was calculated from these images following the methodology detailed in the appendix (Figure S32, Supporting Information). Additionally, weighted MRI of the rat tail vertebrae and lumbar vertebrae of Bama pigs was conducted at various time points post‐surgery using the Achieva 3.0 T MRI system (Philips, Amsterdam, Netherlands). Image evaluation concentrated on the high‐signal‐intensity regions corresponding to the NP in T2‐weighted sagittal images. A panel of more than three orthopedic fellows assessed the degree of IVDD, measuring and evaluating it according to predefined criteria.

### Histological Analysis

Intervertebral disc tissue was extracted from the Co5‐Co6 region of rat tails. The collected caudal vertebral tissues were fixed with PFA and then decalcified using 10% Ethylenediamine tetraacetic acid for 8 weeks. Following decalcification, the tissues were dehydrated, embedded in paraffin, and sectioned to a thickness of 5 µm. To examine morphological changes in the medulla, surrounding fibrous ring, and cartilage endplates, the sections underwent HE, SO, and immunohistochemical staining procedures.

### RNA‐Seq and Data Analysis

Total RNA was extracted from each thymus sample using the RNAmini kit (Qiagen, Germany). The quality of the RNA was assessed through gel electrophoresis and Qubit analysis (Thermo, Waltham, MA, USA). Only samples with high quality (OD260/280 = 1.8 – 2.2, OD260/230 ≥ 2.0, RIN ≥ 6.5, 28S:18S ≥ 1.0, and > 2 µg) were selected for sequencing library construction. Strk‐specific libraries were created using the TruSeq RNA Sample Preparation Kit (Illumina, San Diego, CA, USA) and sequenced on an Illumina Novaseq 6000 sequencer. The quality of the raw data was checked using FastQC v0.11.2 software.

### Statistics and Reproducibility

All experiments and results were independently performed on more than three occasions. Continuous data were presented as mean ± standard deviation and analyzed using GraphPad Prism 9.0 software (GraphPad Prism, USA). Two groups of counting data with a normal distribution were tested using Shapiro‐Wilk's test, and Levene's variance test was used to assess the homogeneity of variance. A two‐tailed t‐test was employed for significance analysis. More than two groups of counting data with a normal distribution were tested by Shapiro‐Wilk's test and homogeneous variance testing was performed by Levene's variance test. Two‐way analysis of variance (ANOVA) was used for significance analysis. All samples were randomly assigned and analyzed together in each experiment. The investigators were blinded to group allocation during data collection and analysis to ensure impartiality.

## Conflict of Interest

The authors declare no conflict of interest.

## Author Contributions

X.W., Z.G., L.C., and J.S. contributed equally to this work. A.M.W., Y.L.Z., Q.P.Q. provided the essential ideas and designed the experiments. X.Z.W., Z.Y.G., L.J.C., J.S., and Y.Y.H. performed most of the experiments and analyzed the data. X.Z.W., Z.Y.G., L.J.C., and J.S. collected the clinical samples. X.Z.W., K.Y.H.K., M.J., Y.M.L., and P.M. drafted the manuscript. A.M.W., Z.Y.L., Q.P.Q., X.Y.W., and X.Q.W. revised the manuscript.

## Supporting information



Supporting Information

## Data Availability

The data that support the findings of this study are available from the corresponding author upon reasonable request.
