# Supplementary material for: Synergistic Modulating of Mitochondrial Transfer and Immune Microenvironment to Attenuate Discogenic Pain
Source: Adv Sci (Weinh). 2025 Mar 27;12(23):2500128. doi: 10.1002/advs.202500128 (PMC12199421; doi:10.1002/advs.202500128)

Supporting Information

**Synergistic Modulating of Mitochondrial Transfer and Immune Microenvironment to Attenuate Discogenic Pain**

*Xinzhou Wang^1,2#^, Zhenyu Guo^1,2#^, Linjie Chen^1#^, Jing Sun^1#^, Kenny Yat Hong Kwan^3^, Morgan Jones^4^, Yan Michael Li^5^, Yangyang Hu^6^, Xueqiang Wang^1^, Pooyan Makvandi^7^, Xiangyang Wang^1^, Qiuping Qian^2^*, Yunlong Zhou^2^* and Aimin Wu^1^**

^1^Department of Orthopaedics, Key Laboratory of Structural Malformations in Children of Zhejiang Province, Key Laboratory of Orthopaedics of Zhejiang Province, Rehabilitation Medicine Center, The Second Affiliated Hospital and Yuying Children’s Hospital of Wenzhou Medical University, Wenzhou, 325000, China.

^2^Zhejiang Engineering Research Center for Tissue Repair Materials, Wenzhou Institute, University of Chinese Academy of Sciences, Wenzhou, 325000, China.

^3^Department of Orthopaedics and Traumatology, Li Ka Shing Faculty of Medicine, The University of Hong Kong, Hong Kong SAR, China.

^4^Spine Unit, The Royal Orthopaedic Hospital, Birmingham B31 2AP, U.K.

^5^Minimally Invasive Brain and Spine Institute, State University of New York Upstate Medical University, 475 Irving Ave, #402, Syracuse, NY 13210, United States.

^6^Department of Orthopedics, The First Affiliated Hospital of Wenzhou Medical University, Wenzhou, 325000, China.

^7^The Quzhou Affiliated Hospital of Wenzhou Medical University, Quzhou People’s Hospital, Quzhou, 324000, China.

^#^These authors contributed equally: Xinzhou Wang, Zhenyu Guo, Linjie Chen, Jing Sun.

*Corresponding Authors’ e-mail: [qianqp@ucas.ac.cn](mailto:qianqp@ucas.ac.cn); [zhouyl@ucas.ac.cn](mailto:zhouyl@ucas.ac.cn); [aiminwu@wmu.edu.cn](mailto:aiminwu@wmu.edu.cn)

**SUPPLEMENTARY FIGURES**


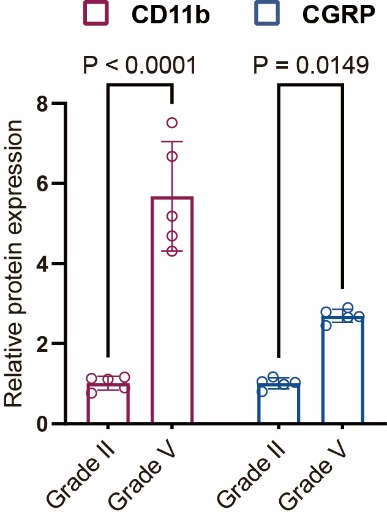


**Figure S1.** Quantification of relative protein expression of CD11b and CGRP (n=5). Data are expressed as mean ± standard deviation.


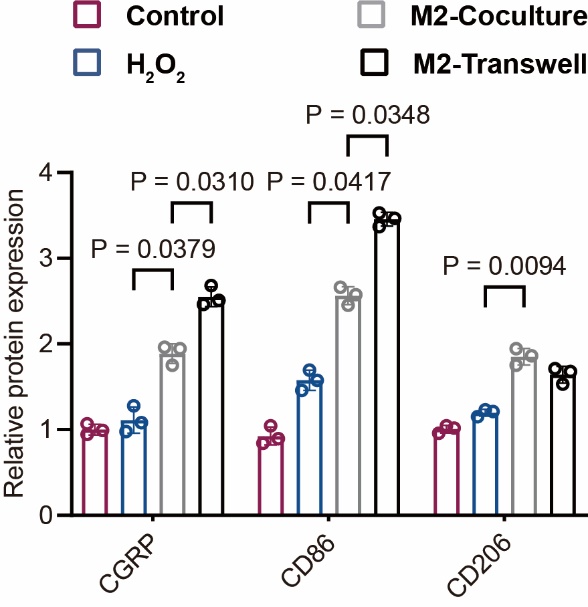


**Figure S2.** Quantification of relative protein expression of CGRP, CD86, and CD206 (n=5). Data are expressed as mean ± standard deviation.


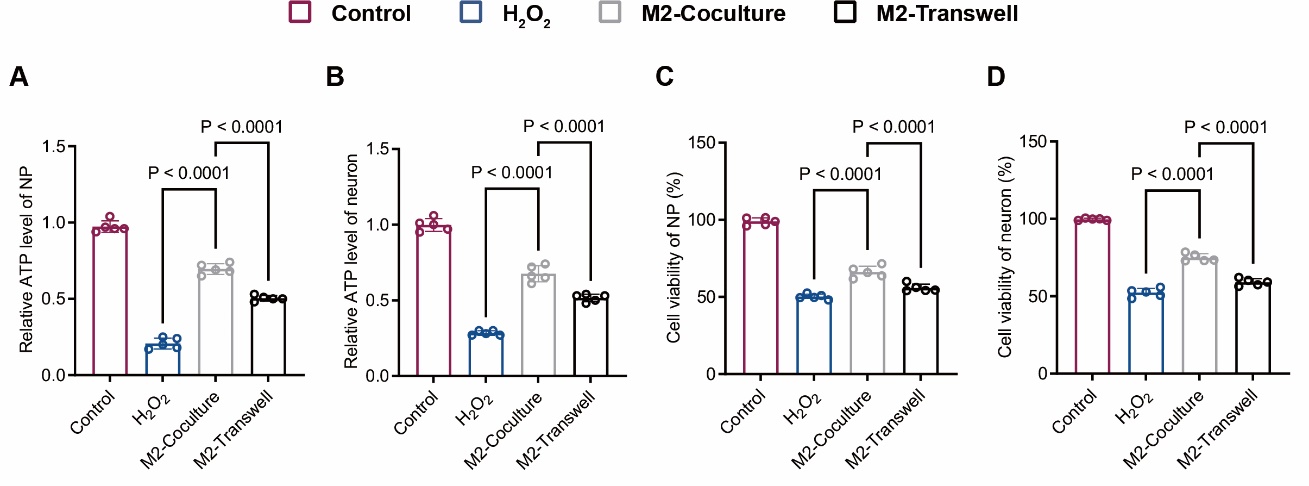


**Figure S3.** (A) Relative ATP amount in NP cells under different treatments (n=3). (B) Relative ATP amount in neurons under different treatments (n=3). (C) Cell viability of NP cells in different treatment conditions (n=3). (D) Cell viability of neurons in different treatment conditions (n=3). Data are expressed as mean ± standard deviation.


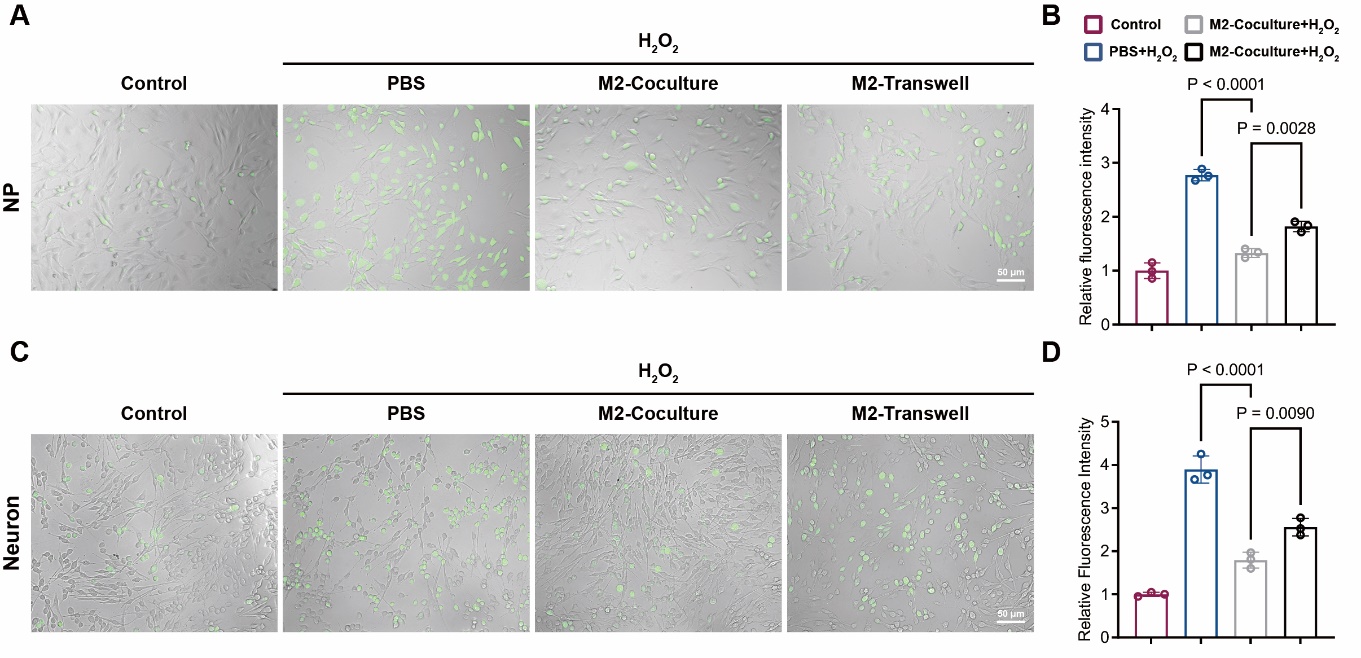


**Figure S4.** (A) Representative DCFH-DA fluorescence staining of NP cells under different treatment conditions (n=3). Scale bar: 50 μm. (B) Quantitative analysis of relative fluorescence intensity under different treatment conditions (n=3). (C) Representative DCFH-DA fluorescence staining of neurons under different treatment conditions (n=3). Scale bar: 50 μm. (D) Cell viability of neurons in different treatment conditions (n=3). Data are expressed as mean ± standard deviation.


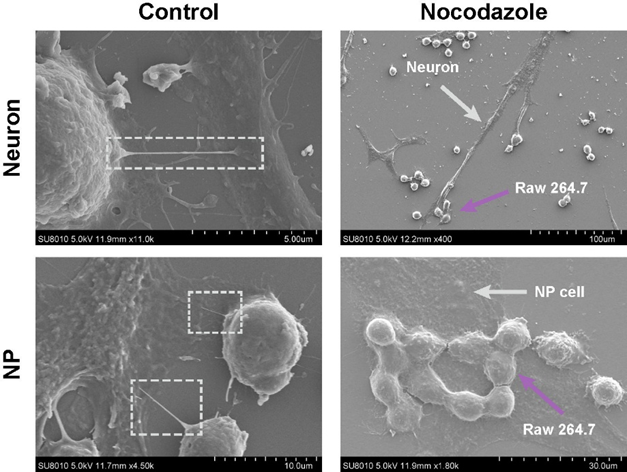


**Figure S5.** SEM images of nanoscale tubular connections between macrophages and NP cells/neurons under different treatment conditions (n=3).


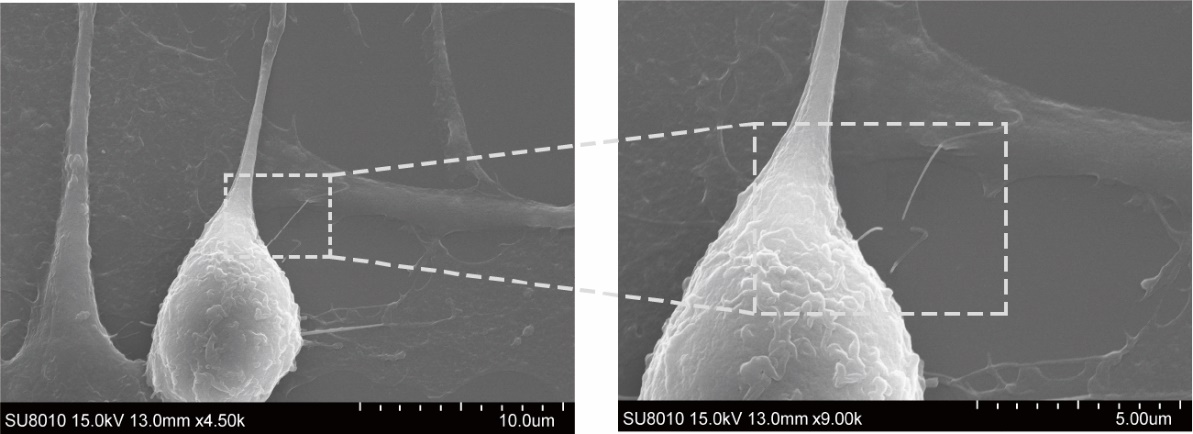


**Figure S6.** SEM images of nanotubes disrupted during imaging.


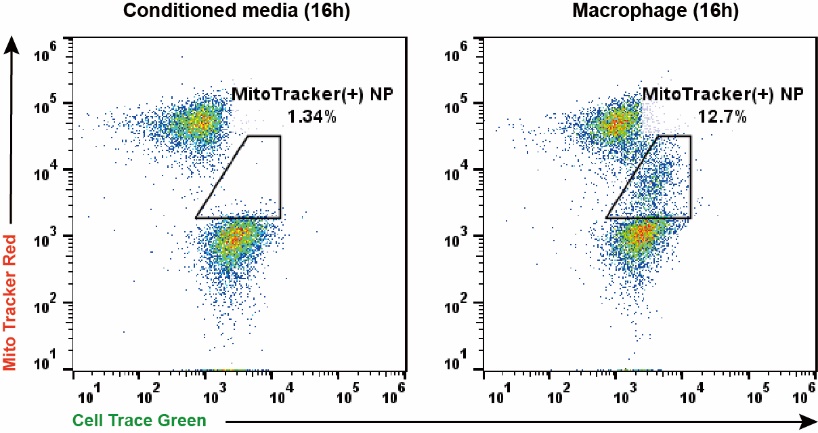


**Figure S7.** Pseudocolor images show the proportion of double-positive cells under different treatment conditions (n=3).


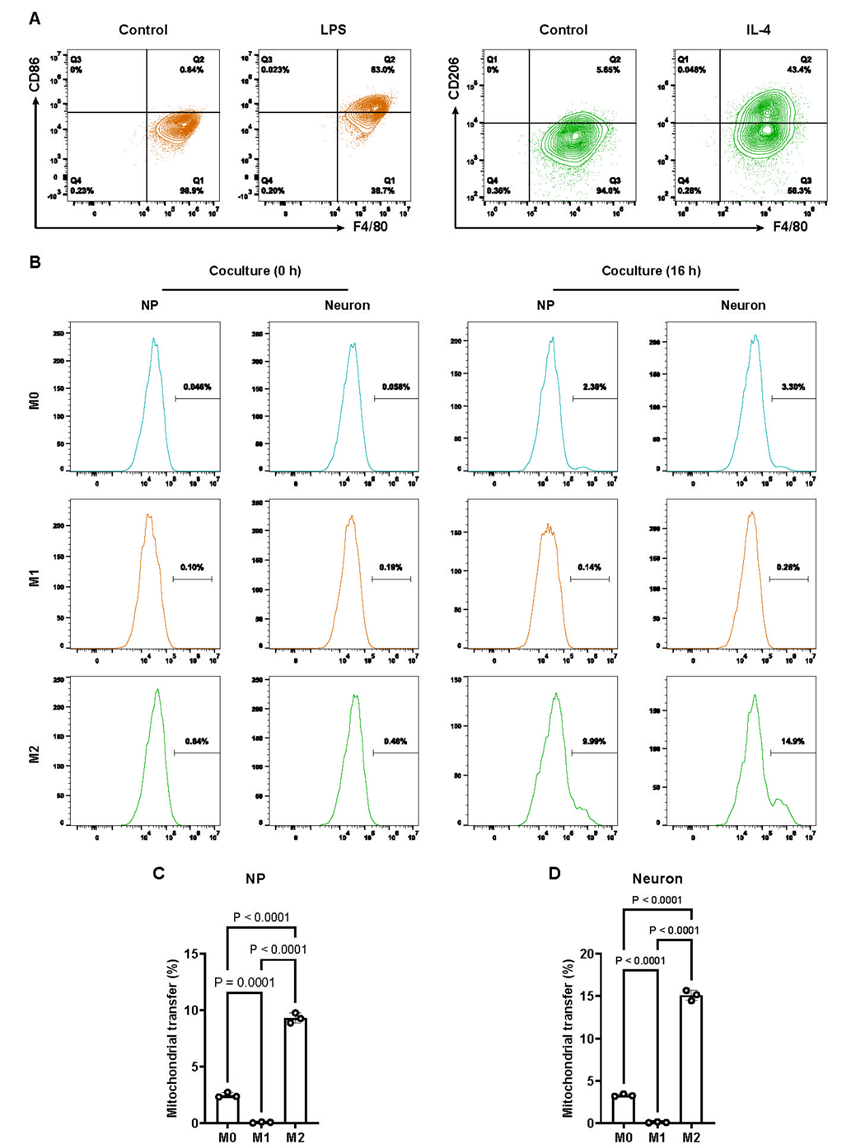


**Figure S8.** (A) Flow cytometry analysis of macrophage polarization. M0 macrophages were polarized to M1 macrophages by LPS stimulation (CD86+, F4/80+ population) and to M2 macrophages by IL-4 stimulation (CD206+, F4/80+ population). Control groups represent untreated macrophages (n=3). (B) Mitochondrial transfer from M0, M1, and M2 macrophages to NP cells and neurons at 0 hours and after 16 hours of coculture (n=3). (C) Quantification of mitochondrial transfer to NP cells by macrophage subtypes (n=3). (D) Quantification of mitochondrial transfer to neurons by macrophage subtypes (n=3). Data are expressed as mean ± standard deviation.


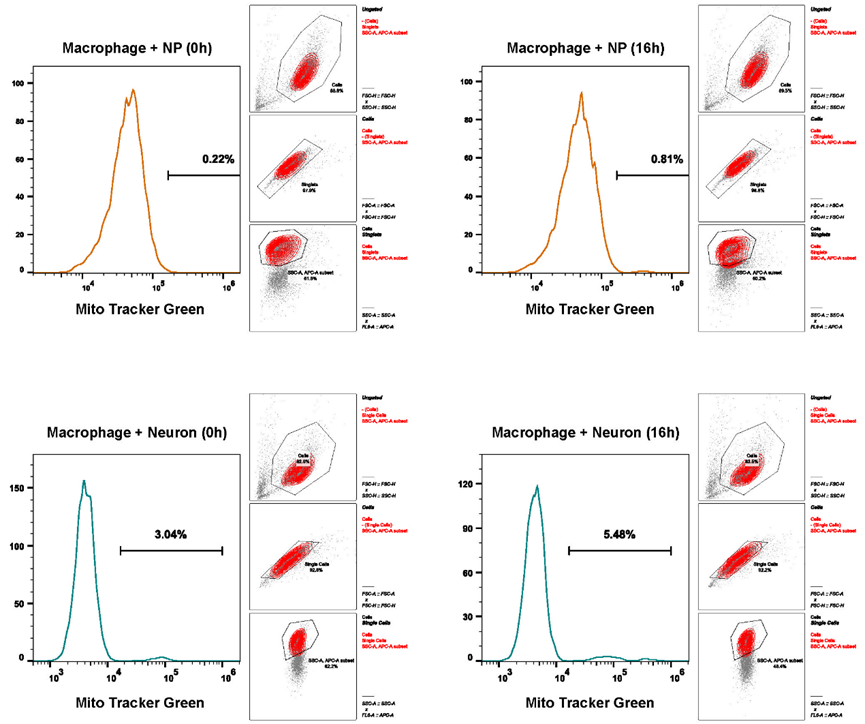


**Figure S9.** Gating strategies of flow-cytometric analysis and histogram of mitochondrial transfer to macrophages by NP cells/neurons (n=3).


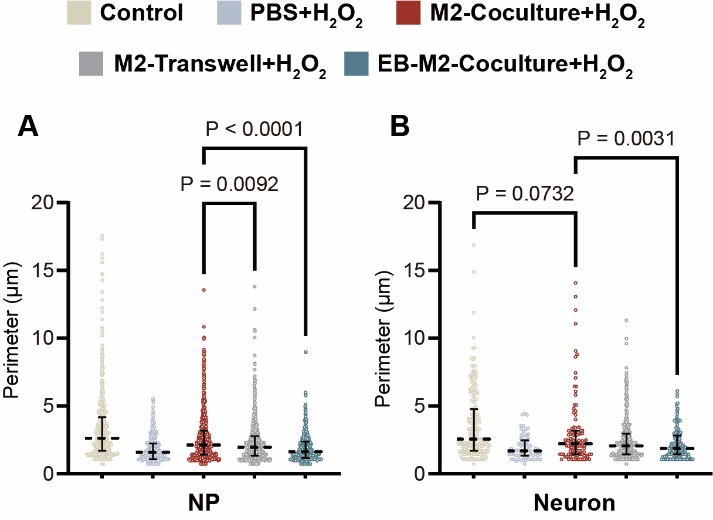


**Figure S10.** (A) ImageJ analysis of the perimeter of mitochondria in NP cells (n=3). (B) ImageJ analysis of the perimeter of mitochondria in NP cells (n=3). Data are expressed as mean ± standard deviation.


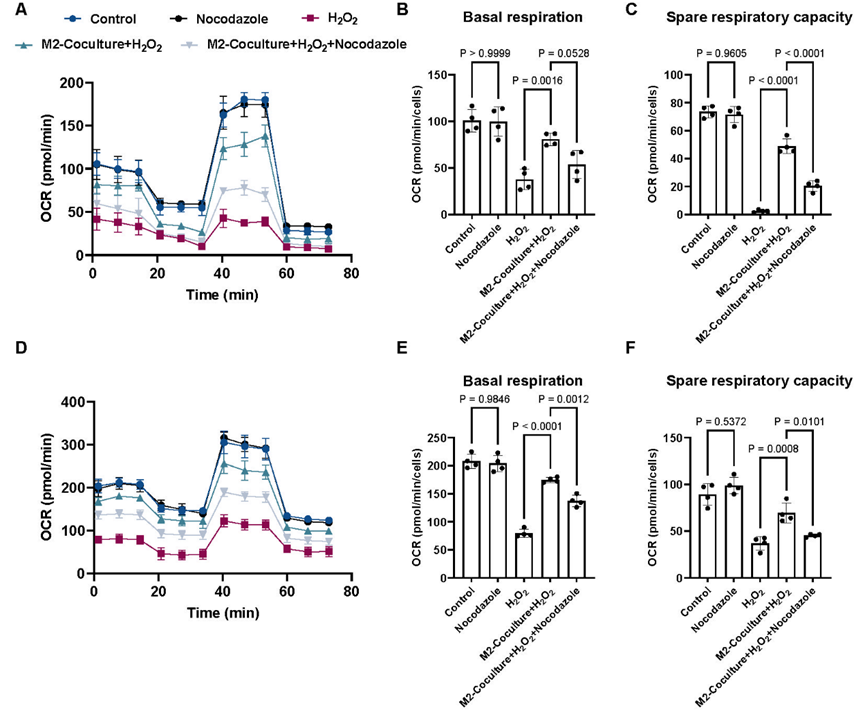


**Figure S11.** (A) The oxygen consumption rate of NP cells under different treatment conditions (n=4). (B) Basal respiration of NP cells under different treatment conditions (n=4). (C) Spare respiratory capacity of NP cells under different treatment conditions (n=4). (D) Oxygen consumption rate of neurons under different treatment conditions (n=4). (E) Basal respiration of NP cells under different treatment conditions (n=4). (F) Spare respiratory capacity of NP cells under different treatment conditions (n=4). Data are expressed as mean ± standard deviation.


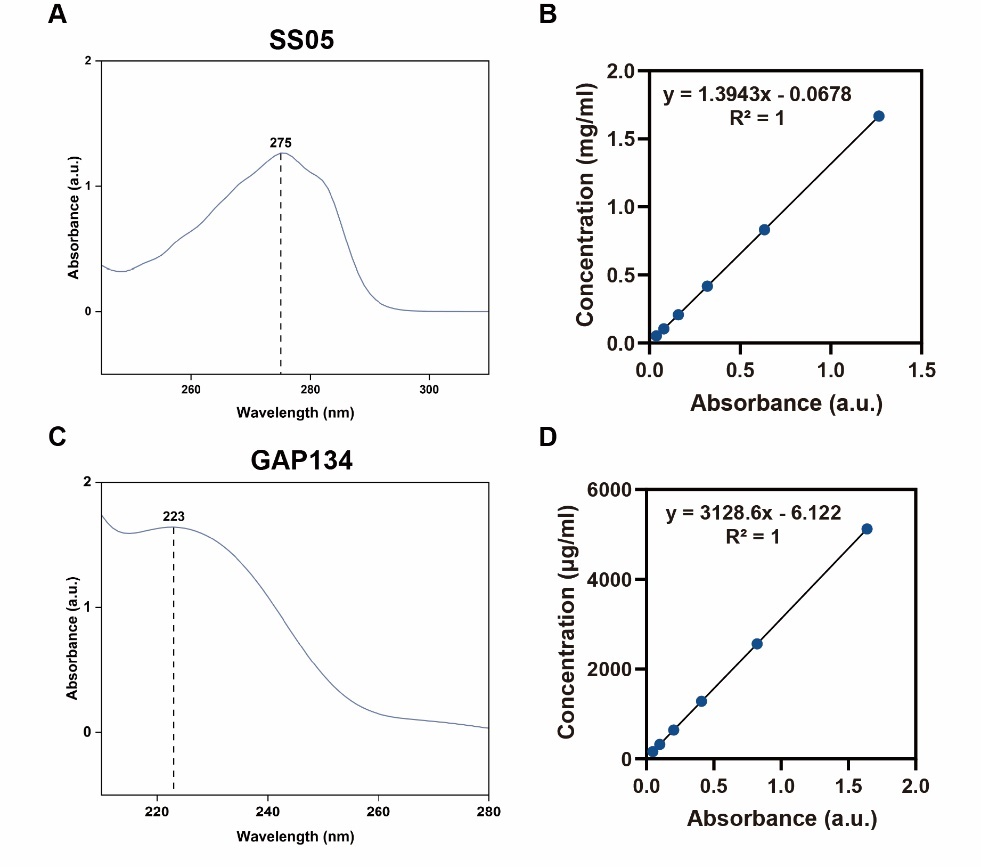


**Figure S12.** (A) UV characteristic absorption peak of SS05. (B) Standard curves for TP04. (C) UV characteristic absorption peak of GAP134. (D) Standard curves for GAP134.


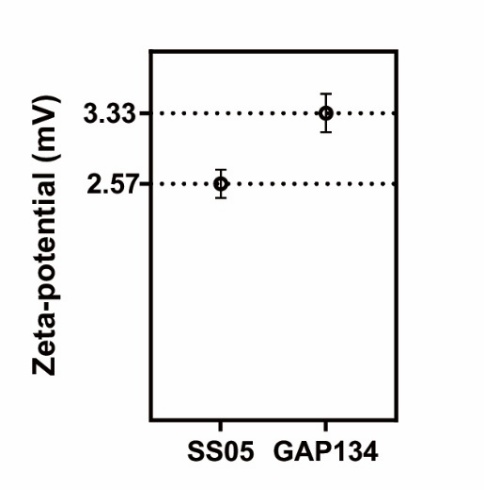


**Figure S13.** Zeta-potential of aqueous solutions of peptides.


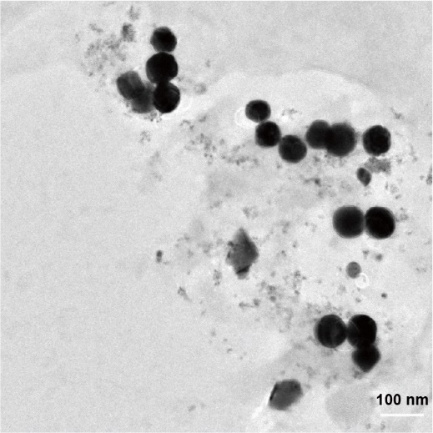


**Figure S14.** TEM images of PGA-Cu-S@G nanoparticles. Scale bar: 100 nm.


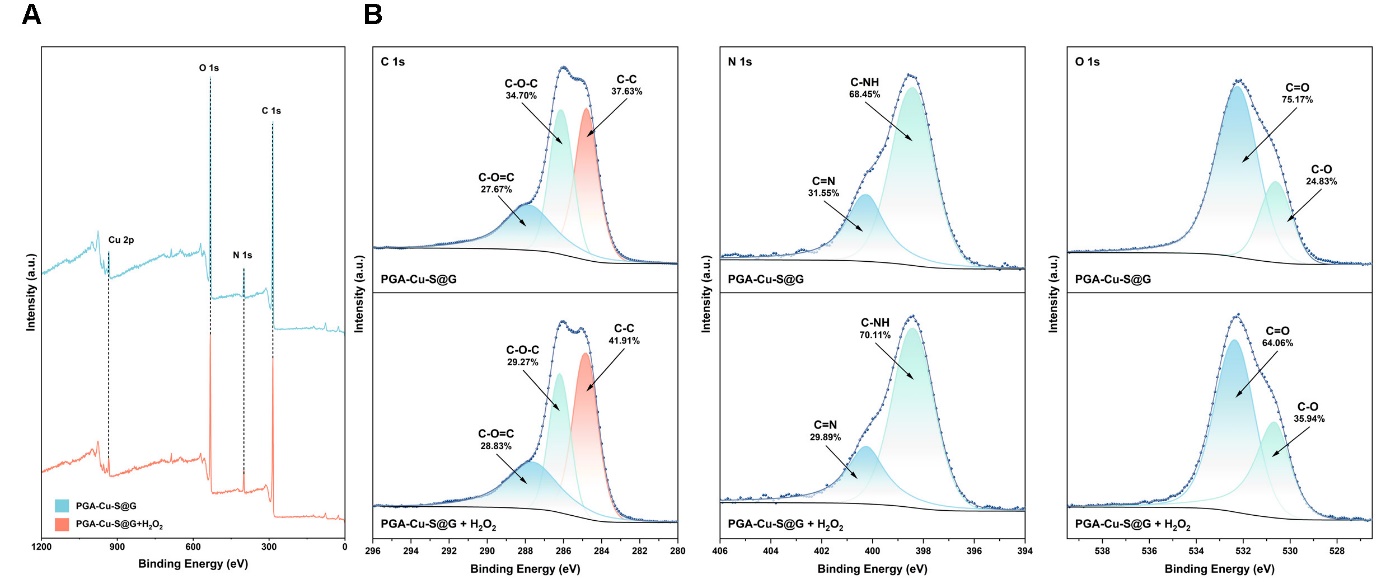


**Figure S15.** Full-scan XPS survey spectra of PGA-Cu-S@G nanoparticles.


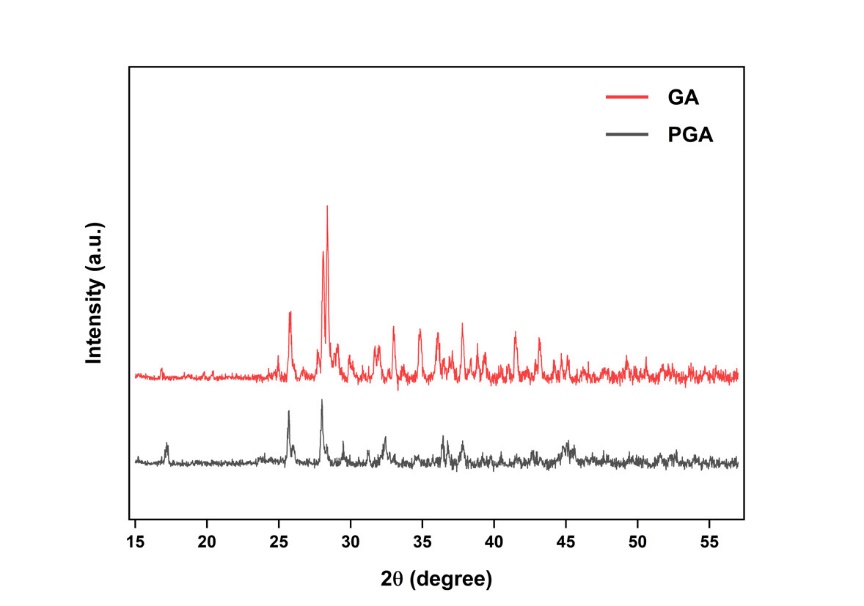


**Figure S16.** (A) XRD of PGA-Cu and PGA-Cu-S@G. (B) XRD of GA and PGA.


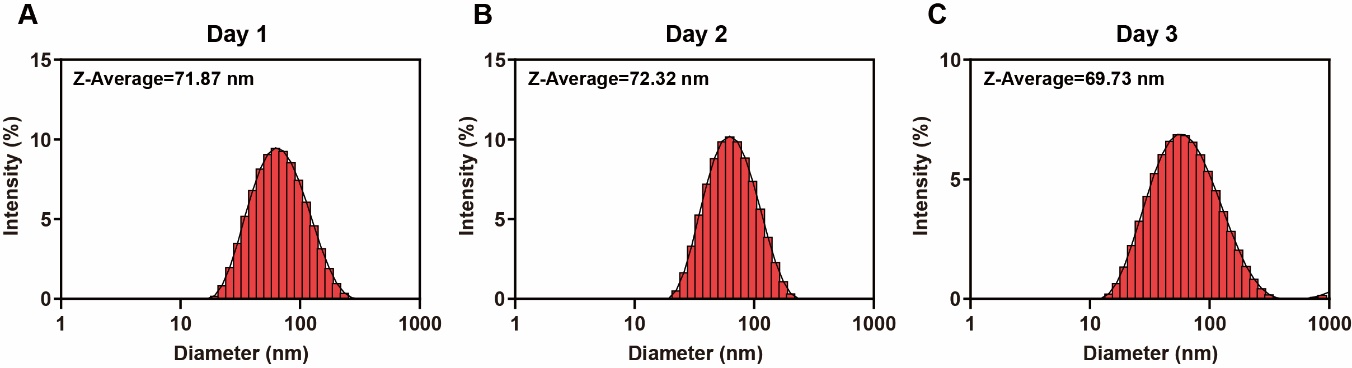


**Figure S17.** DLS size of PGA-Cu-S@G on different days.


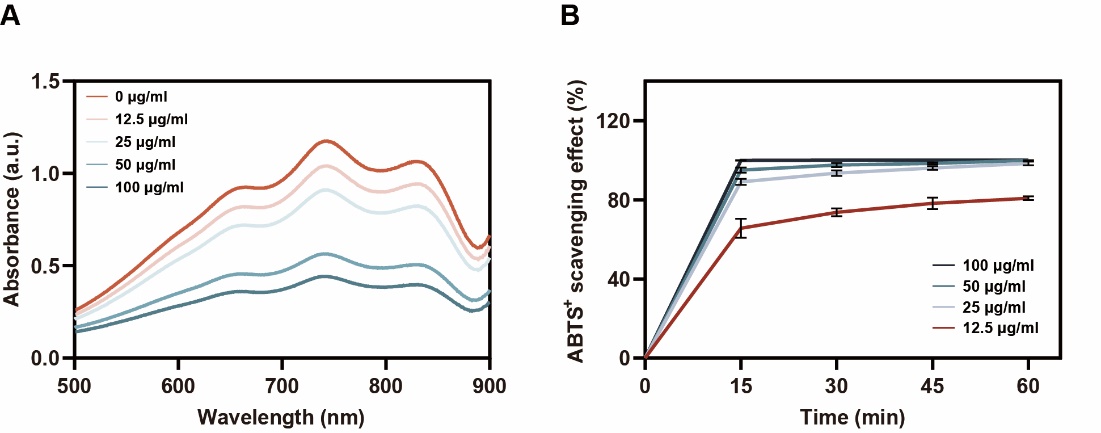


**Figure S18.** (A) UV−vis absorbance spectra showing the ABTS^+^• eliminating activities of PGA-Cu-S@G (B) ABTS^+^• temporal scavenging efficiency of PGA-Cu-S@G with various concentrations (n=3).


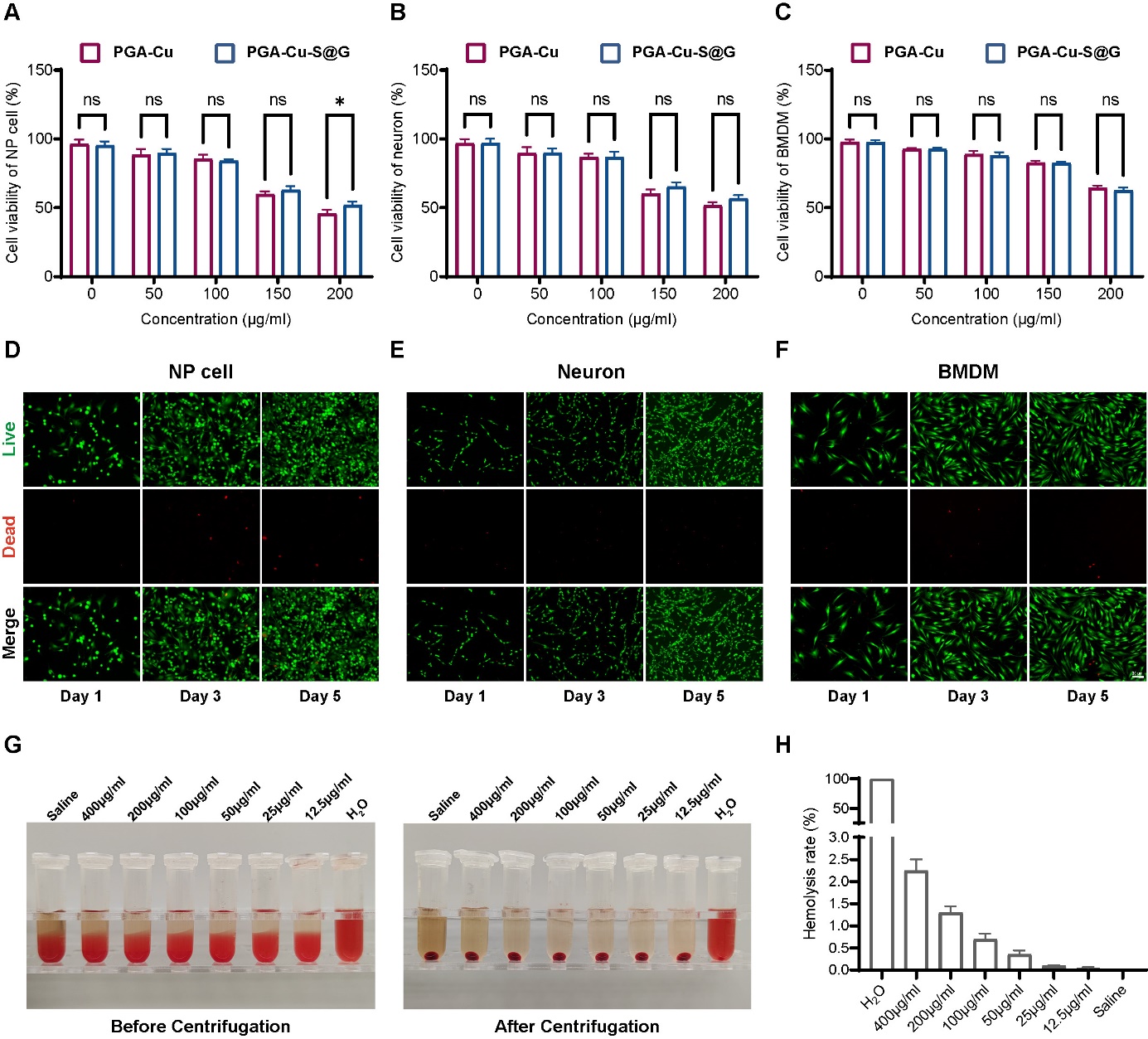


**Figure S19.** (A) Cell viability of NP cells cocultured with PGA-Cu and PGA-Cu-S@G (n=3). (B) Cell viability of neurons cocultured with PGA-Cu and PGA-Cu-S@G (n=3). (C) Cell viability of BMDM cocultured with PGA-Cu and PGA-Cu-S@G (n=3). (D) Live/dead staining of NP cells co-cultured with PGA-Cu-S@G on different days (n=3). Scale bar: 20 μm. (E) Live/dead staining of neurons co-cultured with PGA-Cu-S@G on different days (n=3). Scale bar: 20 μm. (F) Live/dead staining of BMDM co-cultured with PGA-Cu-S@G on different days (n=3). Scale bar: 20 μm. (G) Digital photographs of different hemolysis samples before and after centrifugation. (H) Hemolysis ratios of the PGA-Cu-S@G with different concentrations (n=3). *p < 0.05; **p < 0.01; ***p < 0.001; ****p < 0.0001; ns, not significant. Data are expressed as mean ± standard deviation.


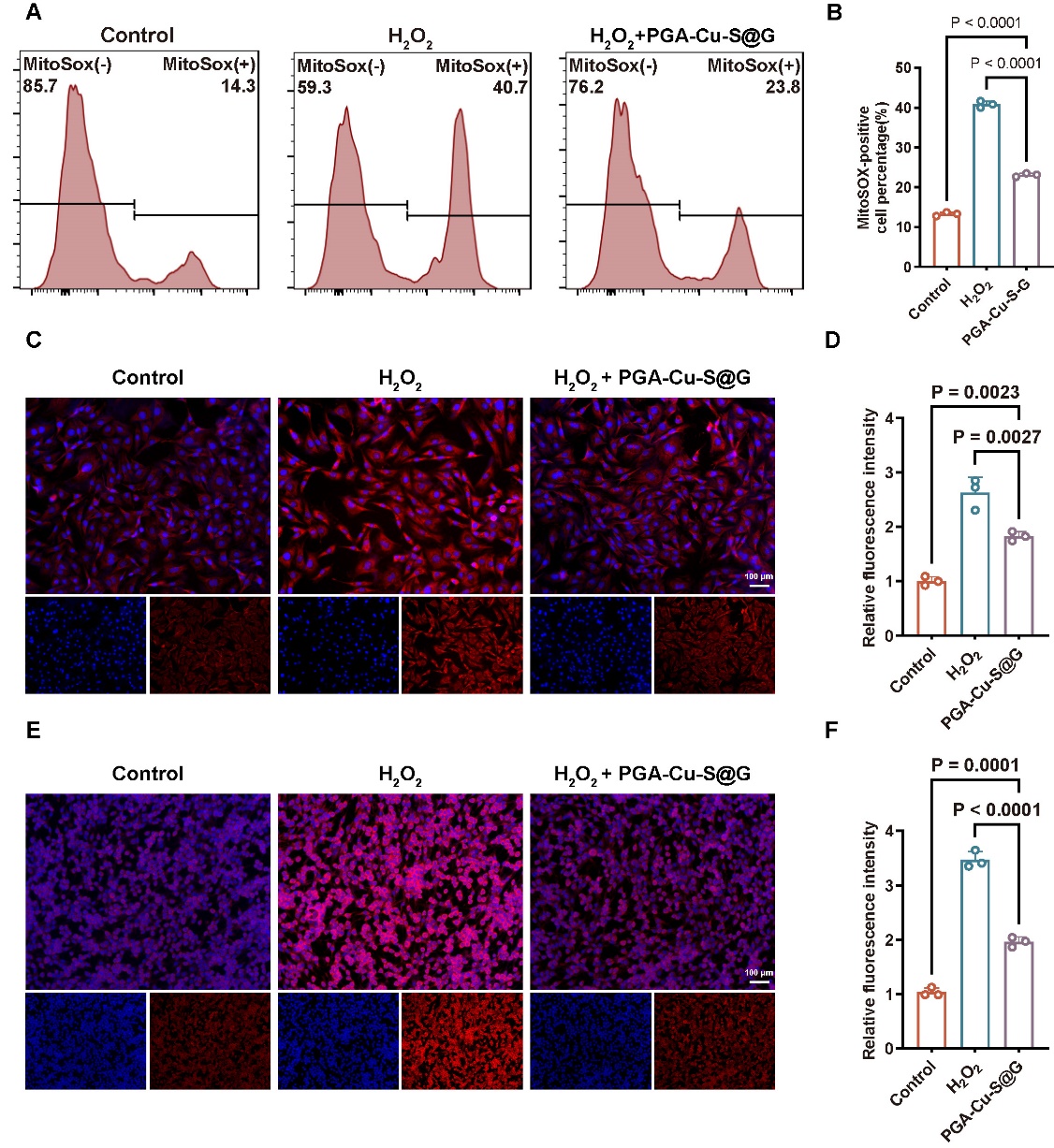


**Figure S20.** (A) Flow cytometry analysis of Mito Sox fluorescence intensity of macrophages (n=3). (B) Quantitative analysis of Mito Sox (+) cell percentage (n=3). (C) Representative fluorescence images of Mito-Sox staining of NP cells under different treatment conditions (n=3). Scale bar: 100 μm. (D) Quantitative analysis of Mito Sox fluorescence intensity (n=3). (E) Representative fluorescence images of Mito-Sox staining of NP cells under different treatment conditions (n=3). Scale bar: 100 μm. (F) Quantitative analysis of Mito-Sox fluorescence intensity (n=3). Data are expressed as mean ± standard deviation.


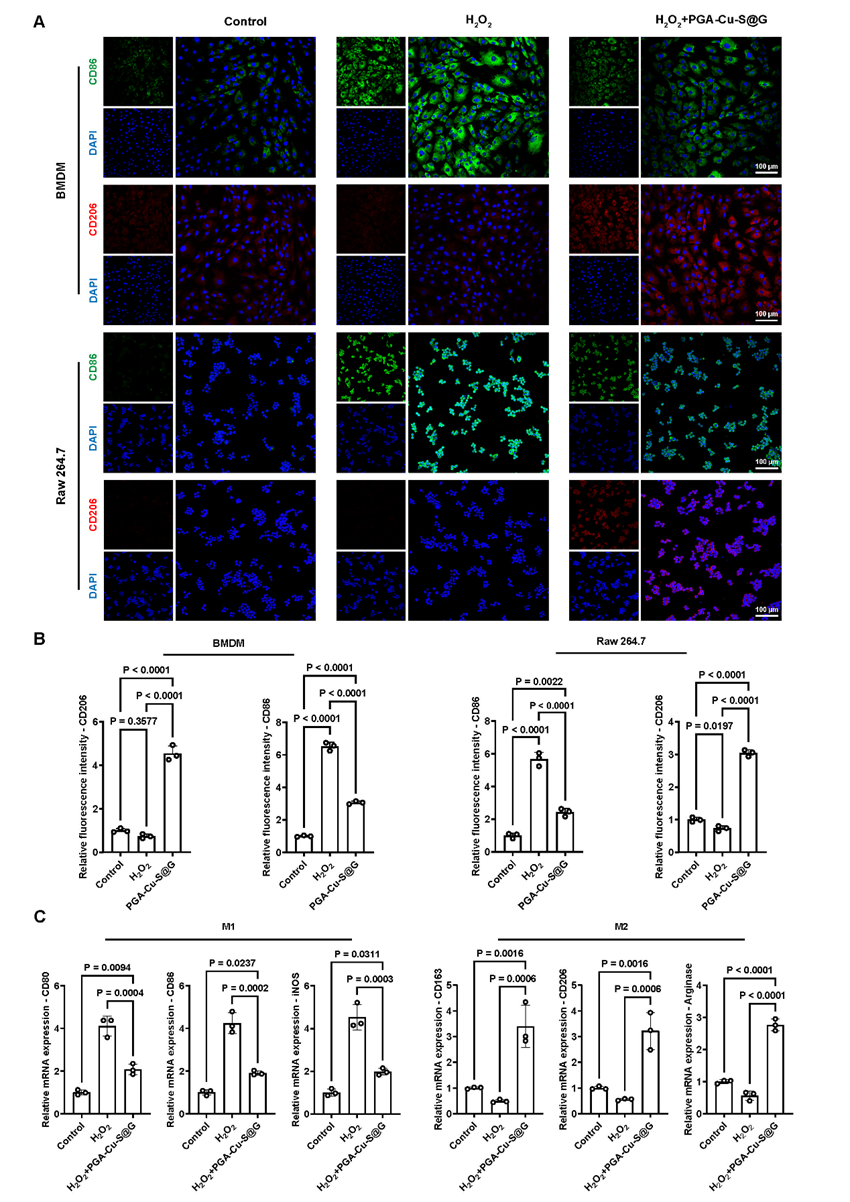


**Figure S21.** (A) Representative fluorescent images of CD86 and CD206 staining under different treatment conditions (n=3). (B) Quantitative analysis of CD86 and CD206 relative fluorescence intensity (n=3). (C) Quantitative qRT-PCR analysis of macrophage polarization markers (n=3). Data are expressed as mean ± standard deviation.


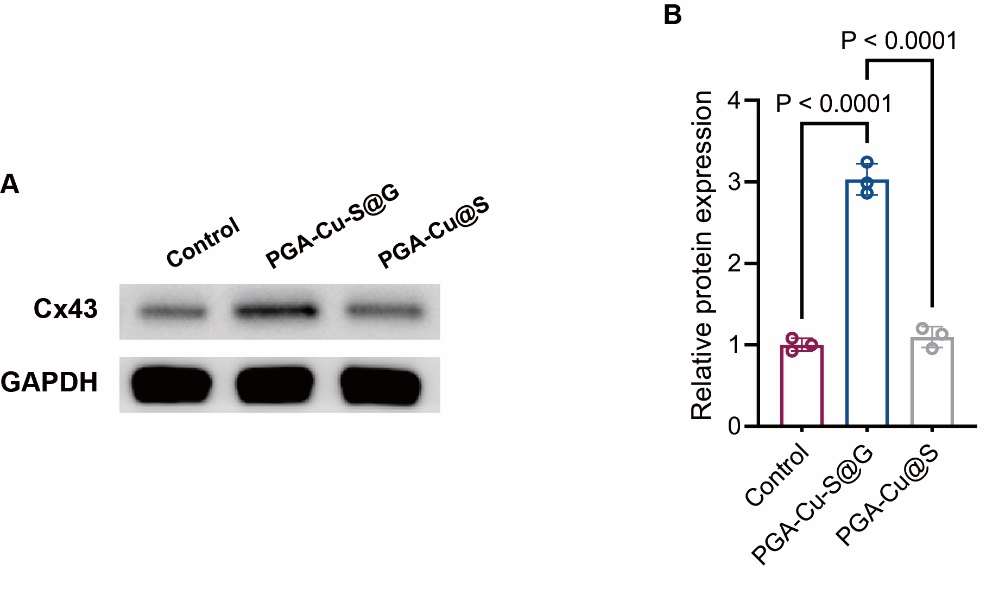


**Figure S22.** (A) Western blot images of Cx43 (n=3). (B) Quantification of relative protein expression of Cx43 (n=3). Data are expressed as mean ± standard deviation.


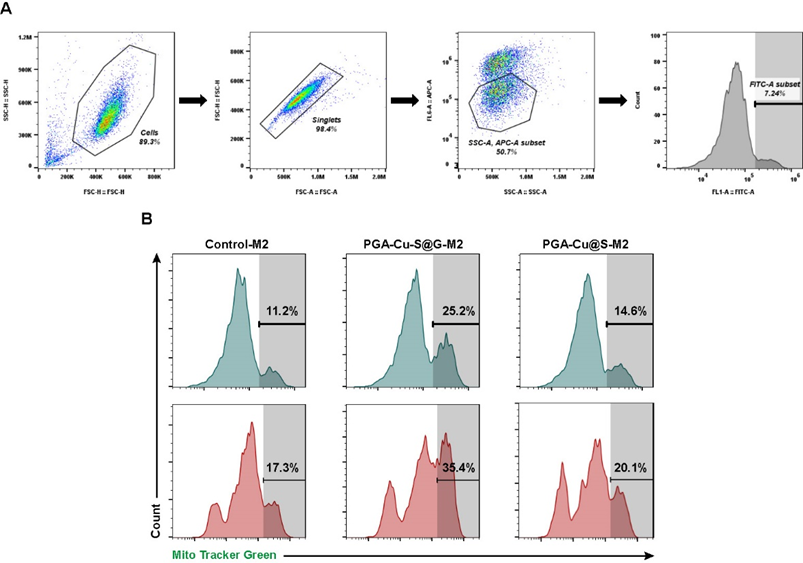


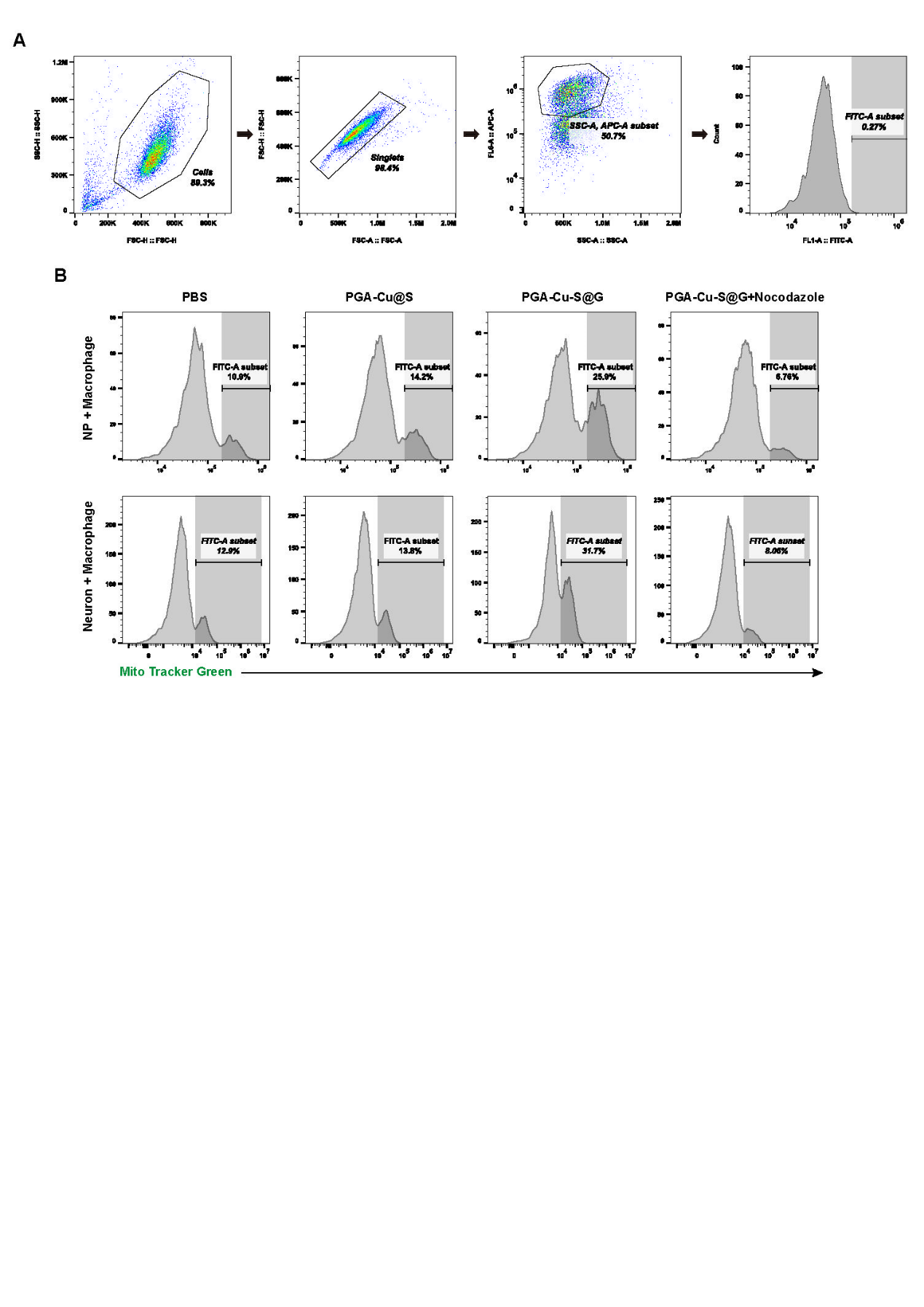


**Figure S23.** (A) Gating strategies of flow-cytometric analysis. (B) Histogram of Mito Tracker Green-positive subset under different treatment conditions (n=3). Data are expressed as mean ± standard deviation.


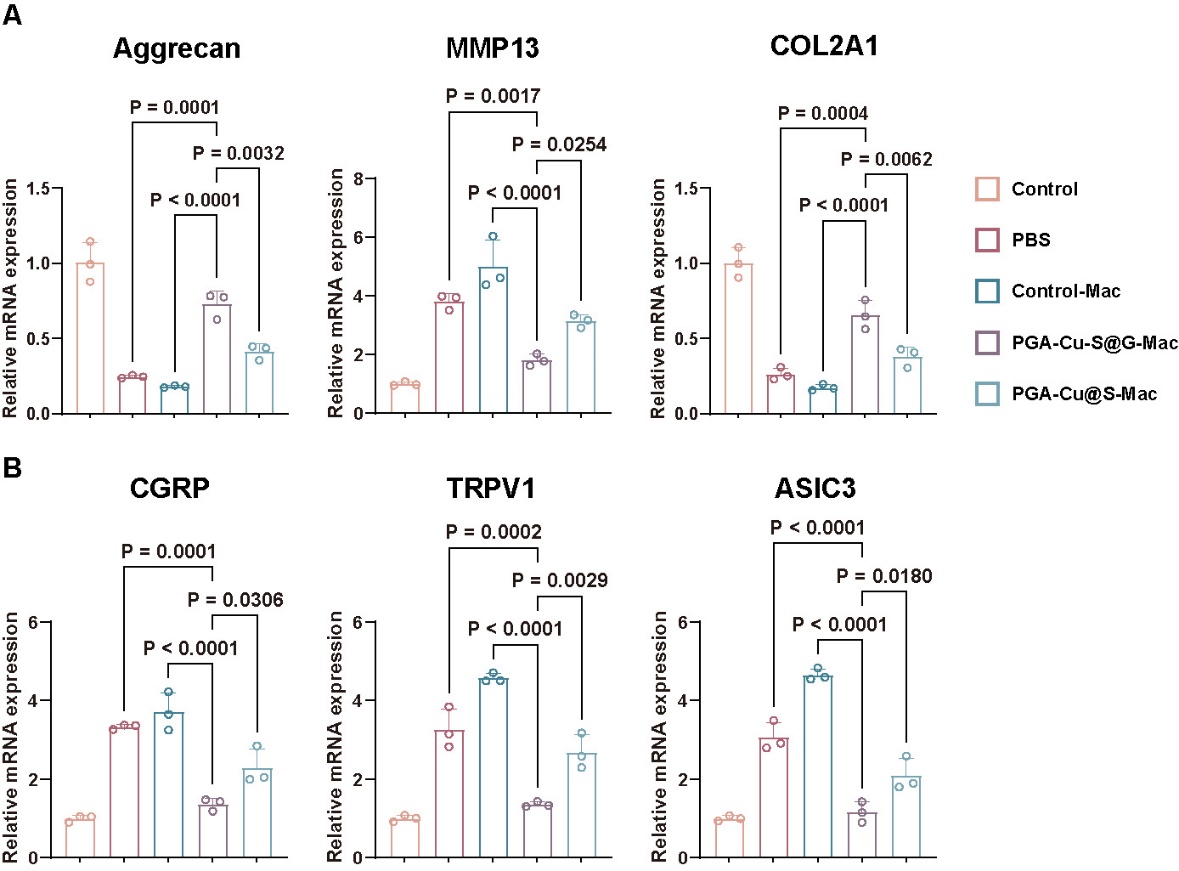


**Figure S24.** (A) Quantitative PCR analysis of representative extracellular matrix-related genes in NP cells (n=3). (B) Quantitative qRT-PCR analysis of representative pain signaling-related genes in neurons (n=3). Data are expressed as mean ± standard deviation.


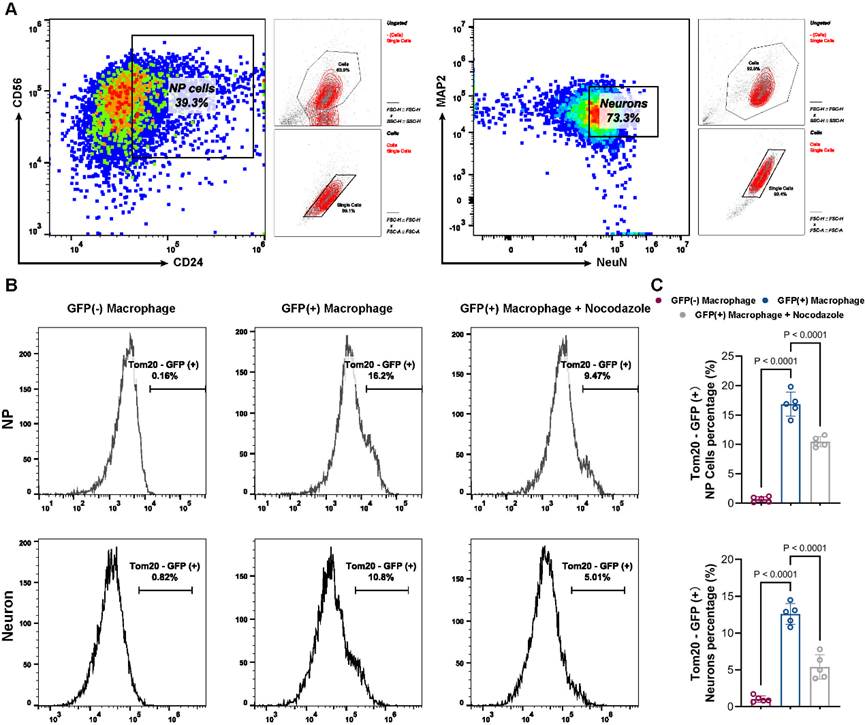


**Figure S25.** (A) Gating strategies for identification of NP cells and neurons by flow cytometry. (B) Histogram of Tom20-GFP-positive subset under different treatment conditions (n=5). (C) Quantification of mitochondrial transfer (n=5). Data are expressed as mean ± standard deviation.


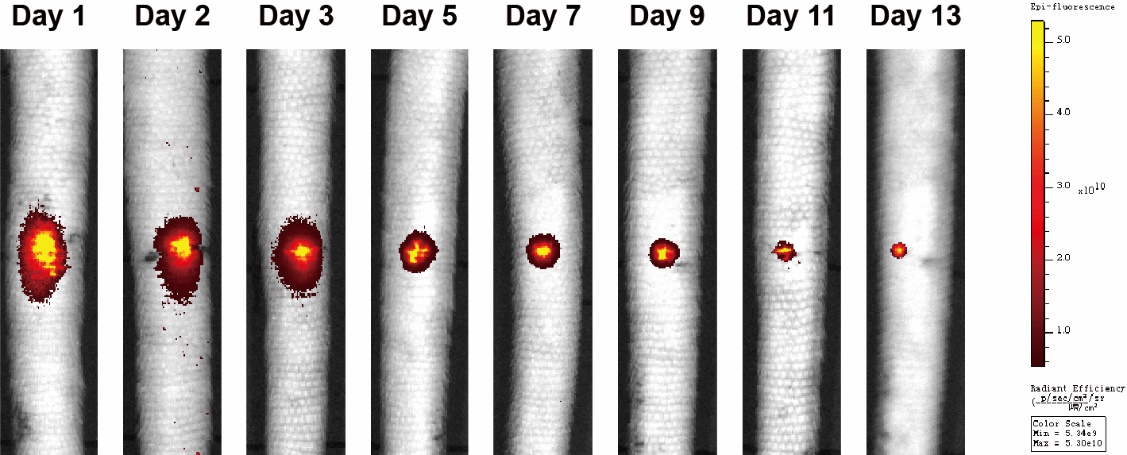


**Figure S26.** *In vivo* imaging of PGA-Cu-S@G in rat coccygeal vertebrae (n=5).


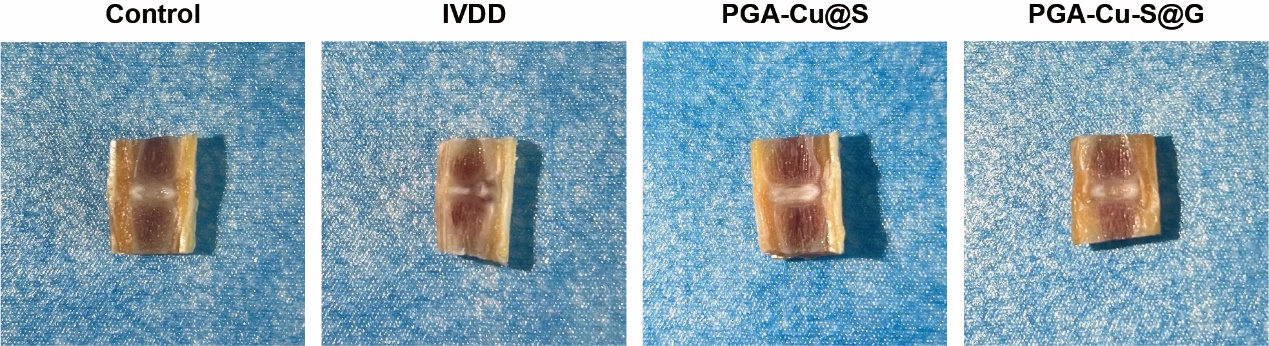


**Figure S27.** Digital photographs of rat caudal intervertebral disc (n=5).


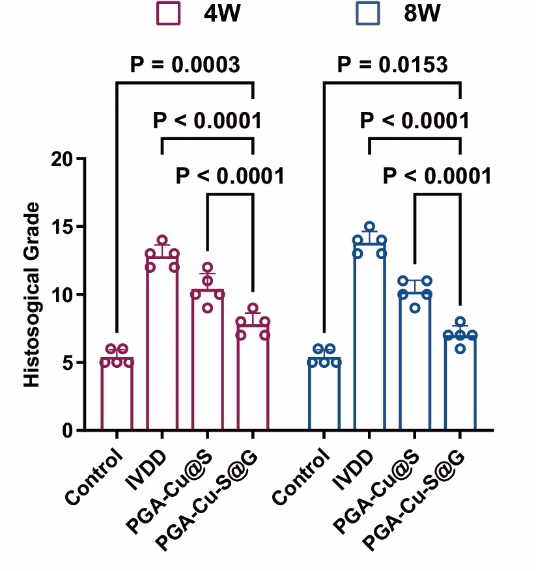


**Figure S28.** Quantification of histological grade (n=5). Data are expressed as mean ± standard deviation.


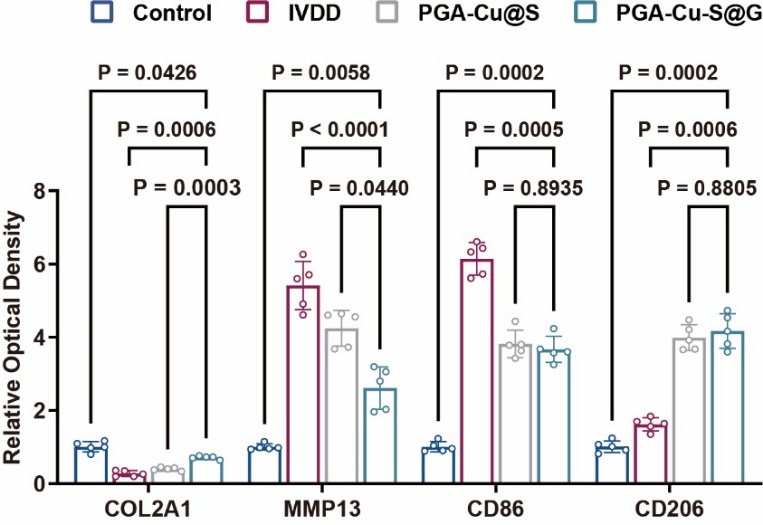


**Figure S29.** Quantitative analysis of immunohistochemistry (n=5). Data are expressed as mean ± standard deviation.


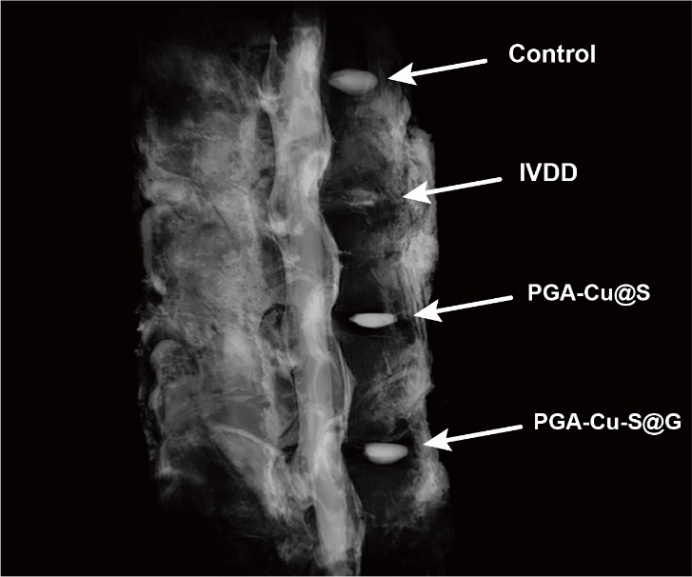


**Figure S30.** MRI images of the lumbar spine in Bama pigs after different treatments (n=3).


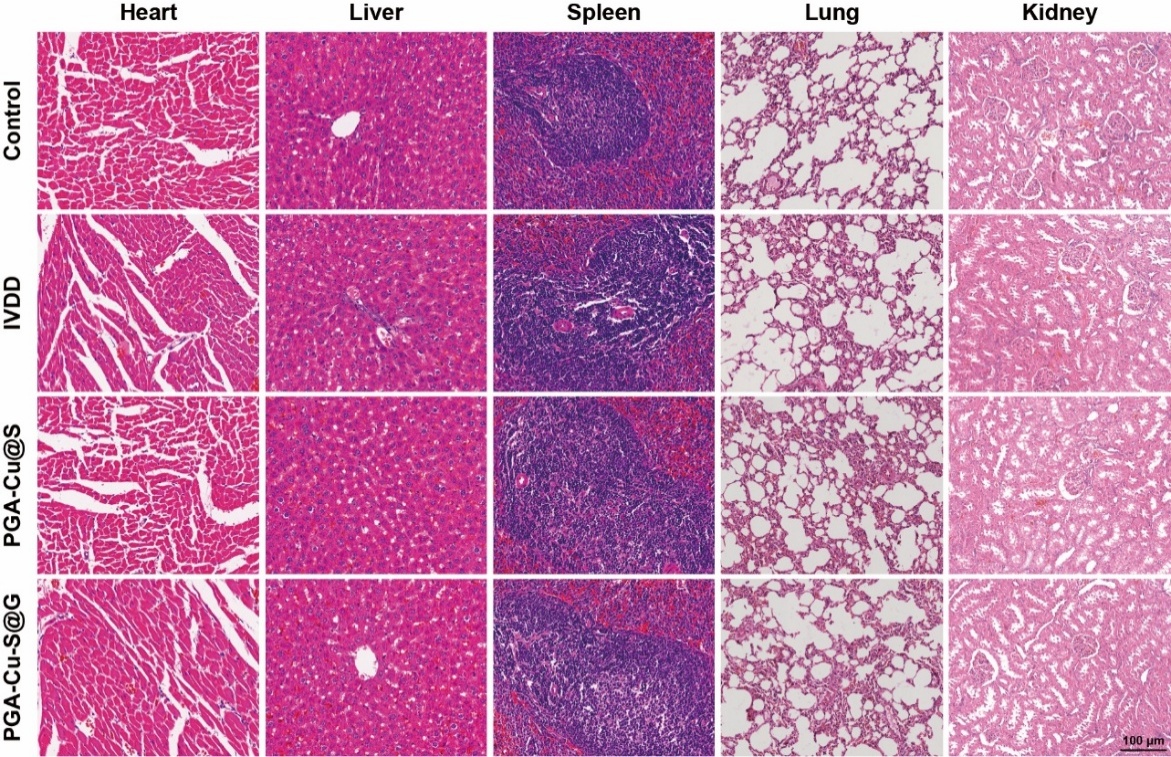


**Figure S31.** HE staining images of various organs at 8 weeks after local injection of PGA-Cu@S and PGA-Cu-S@G (n=5). Scale bar: 100 μm.


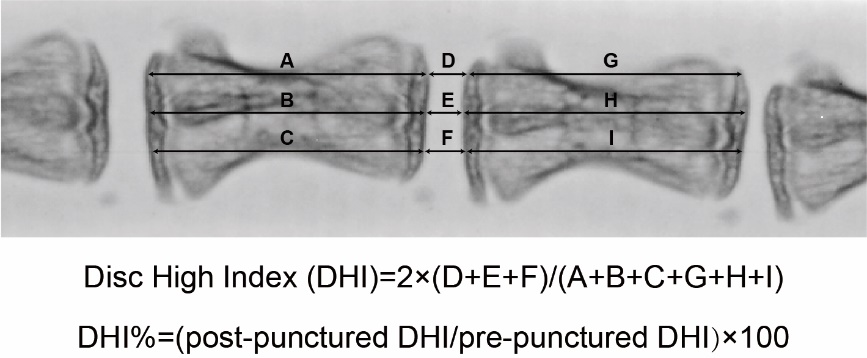


**Figure S32.** Measurement of disc height index (DHI) for intervertebral space height in X-ray images.

**SUPPLEMENTARY TABLES**

**Table S1.** ICP-MS analysis of PGA-Cu-S@G.


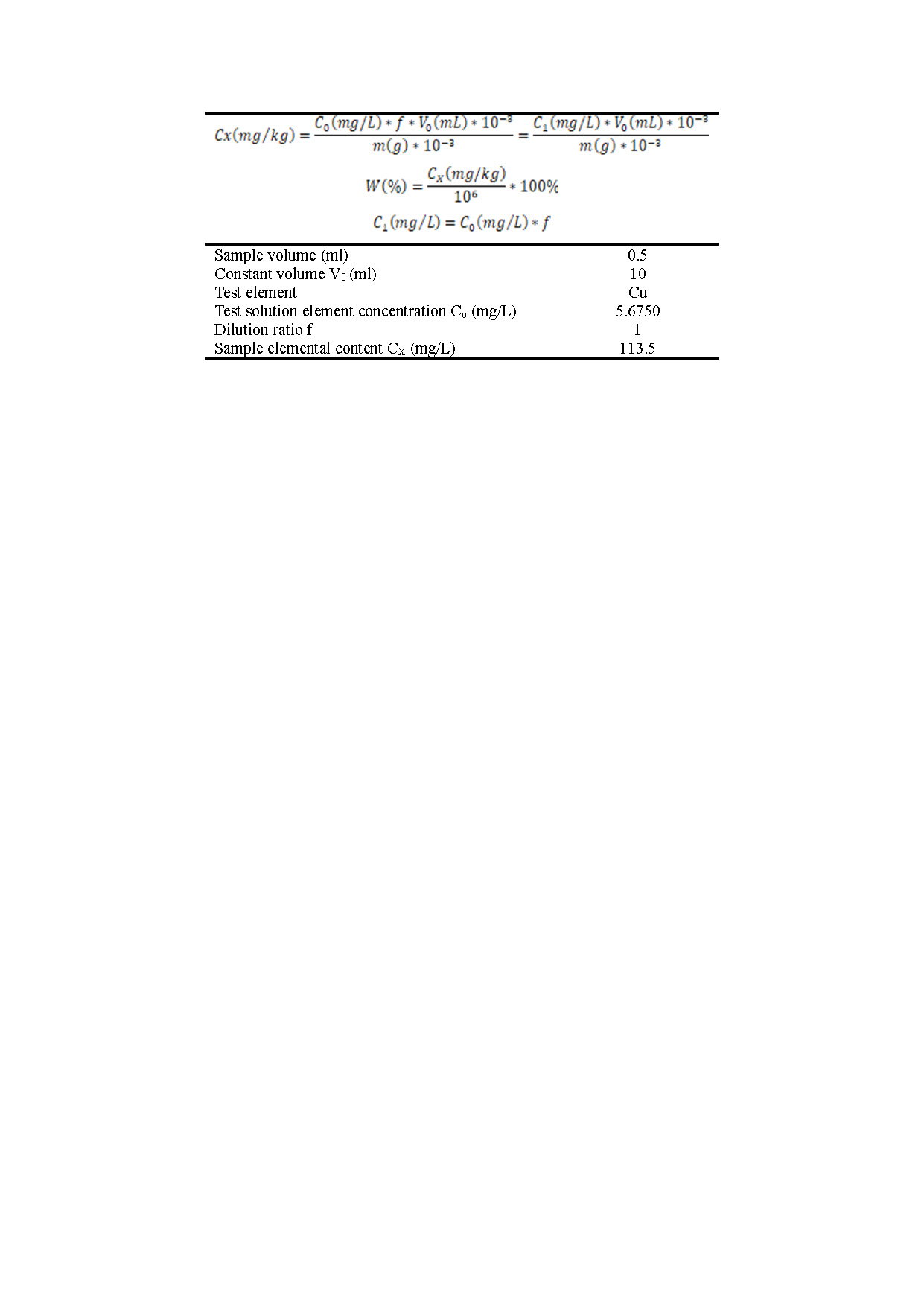


**Table S2.** Pfirrmann classification.


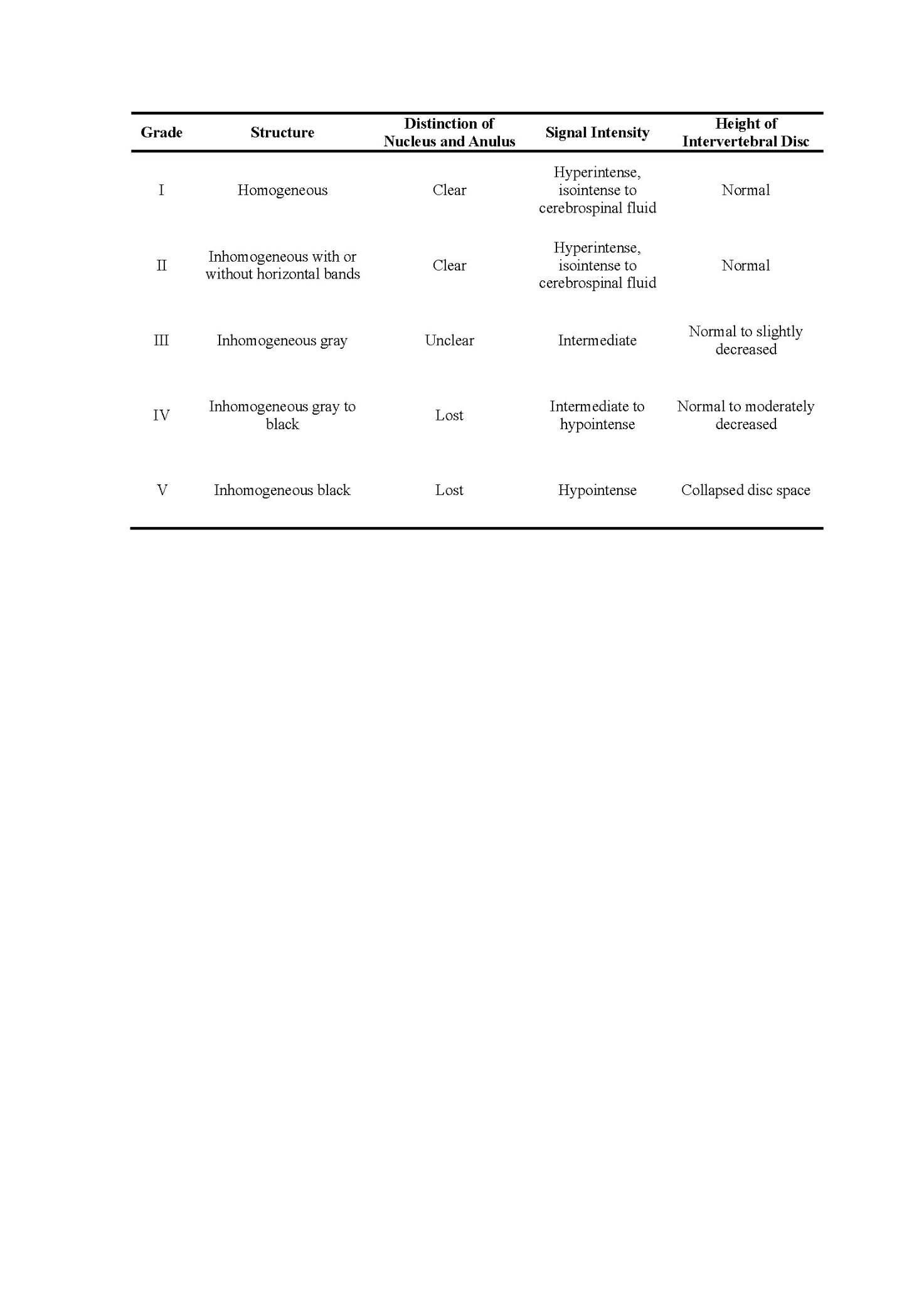


**Table S3.** Histological grading scale.


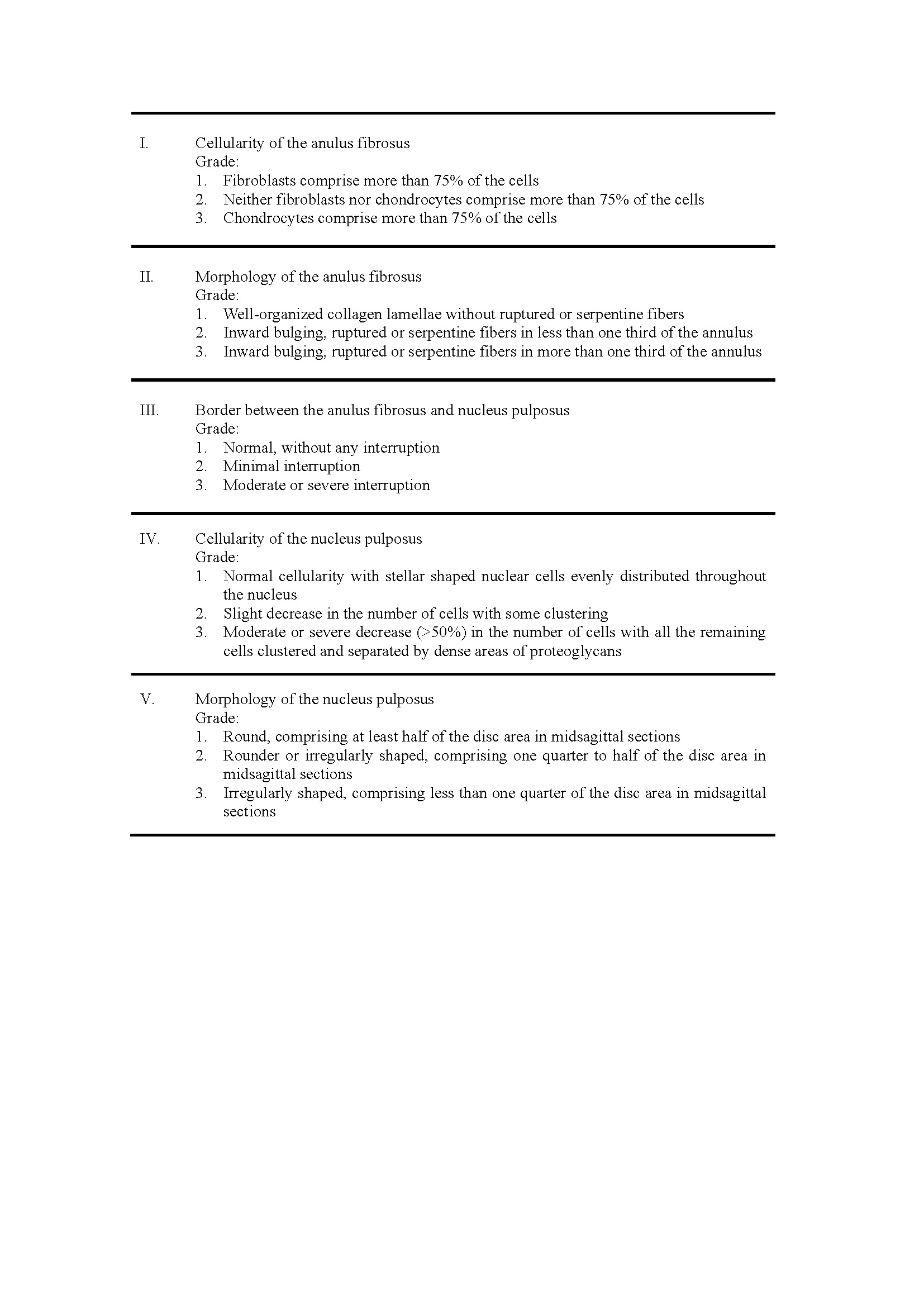


**Table S4.** Primary antibodies used in this study.


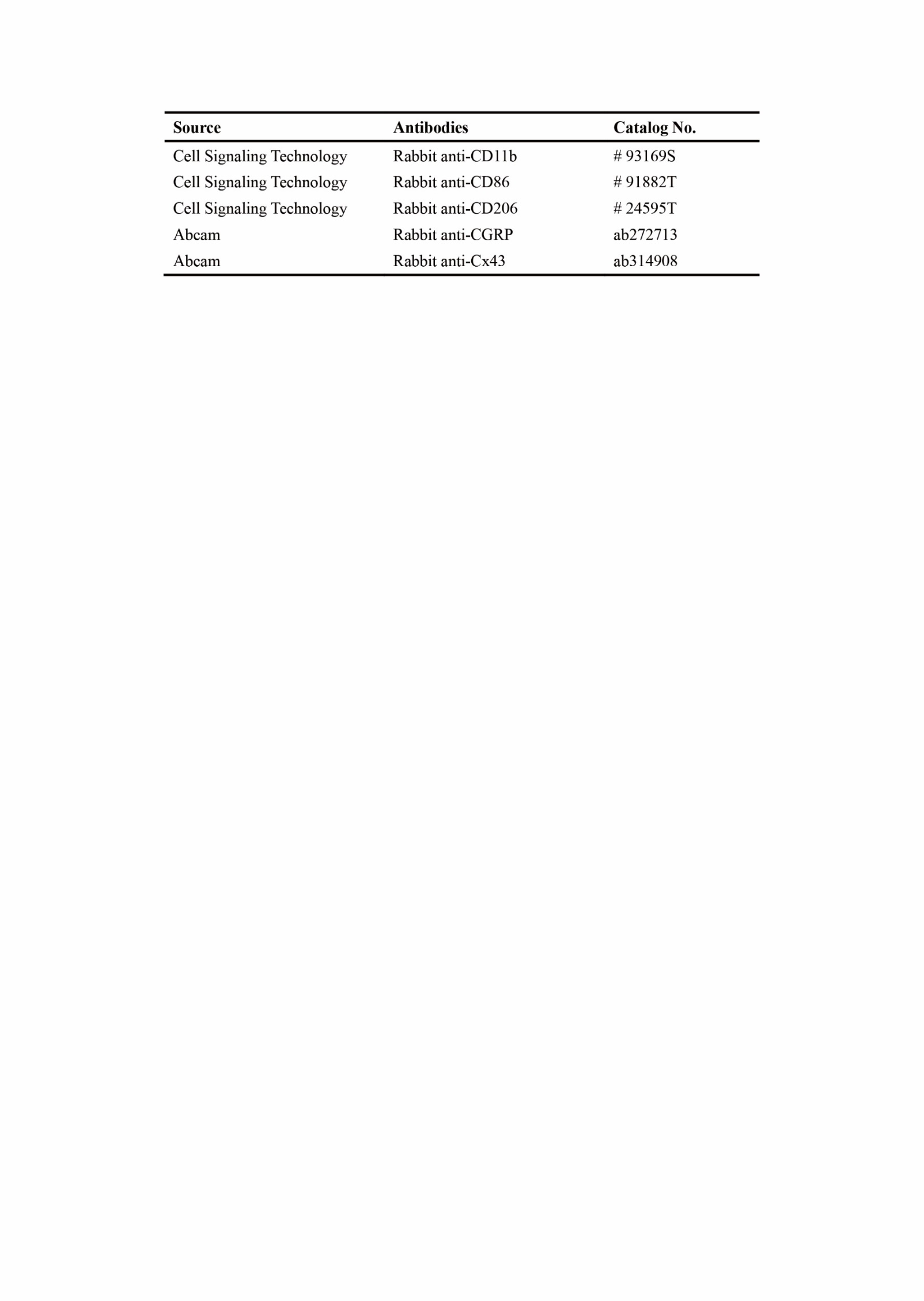


**Table S5.** Primers used for qPCR.


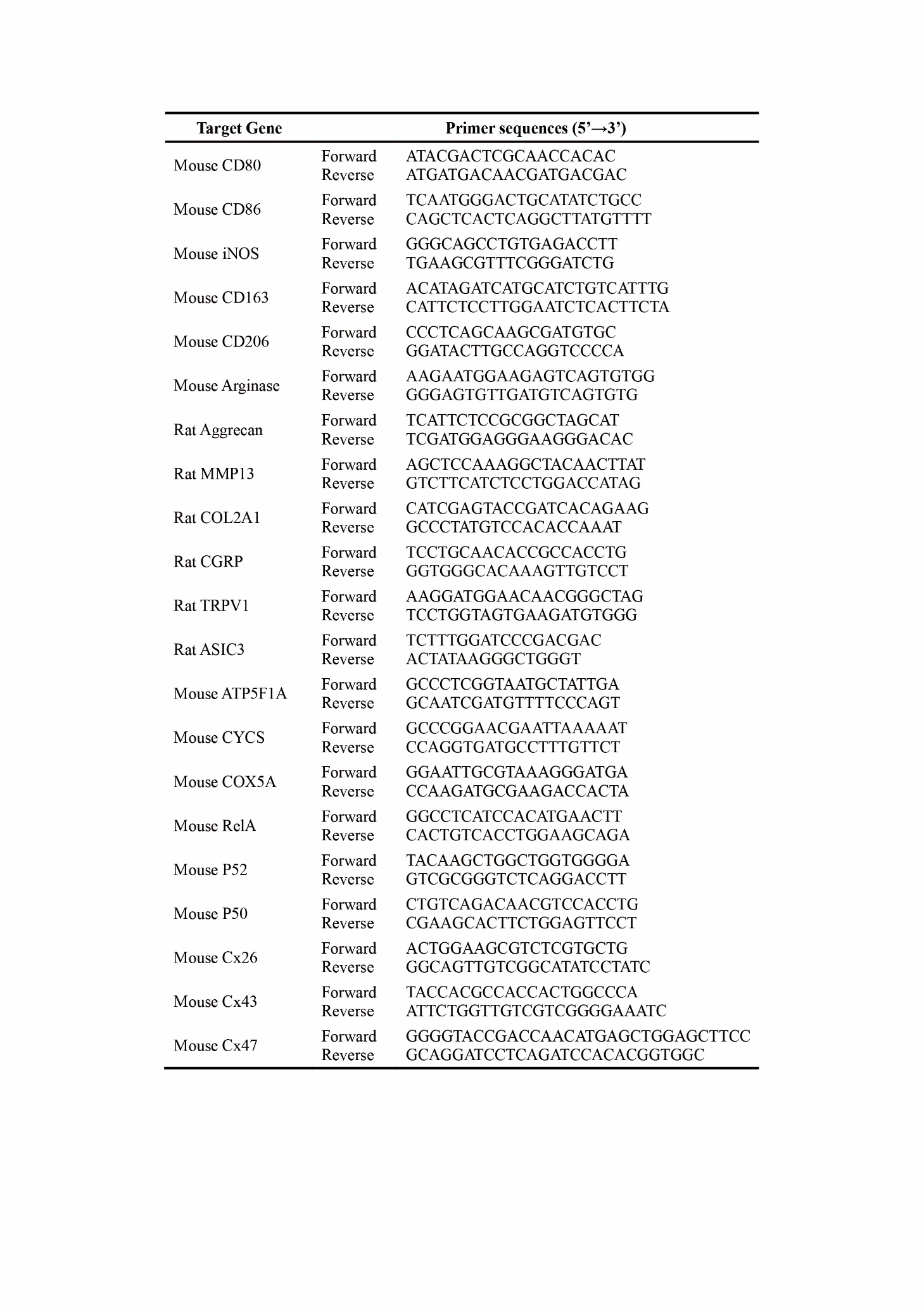

Supplement: Supplementary file 1 — Supporting Information [file ADVS-12-2500128-s001.docx]
